# Conformal geodesics and the evolution of spacetimes with positive Cosmological constant

**DOI:** 10.1098/rsta.2023.0040

**Published:** 2024-03-04

**Authors:** Marica Minucci

**Affiliations:** School of Mathematical Sciences, Queen Mary, University of London, Mile End Road, London E1 4NS, UK

**Keywords:** conformal geodesics, conformal Gaussian gauge systems, positive Cosmological constant, nonlinear stability, de Sitter-like spacetime, Schwarzschild–de Sitter spacetime

## Abstract

This article provides a discussion on the construction of conformal Gaussian gauge systems to study the evolution of solutions to the Einstein field equations with positive Cosmological constant. This is done by means of a gauge based on the properties of conformal geodesics. The use of this gauge, combined with the extended conformal Einstein field equations, yields evolution equations in the form of a symmetric hyperbolic system for which standard Cauchy stability results can be employed. This strategy is used to study the global properties of de Sitter-like spacetimes with constant negative scalar curvature. It is then adapted to study the evolution of the Schwarzschild–de Sitter spacetime in the static region near the conformal boundary. This review is based on Minucci *et al*. 2021 *Class. Quantum Grav*. **38**, 145026. (doi:10.1088/1361-6382/ac0356) and Minucci *et al*. 2023 *Class. Quantum Grav*. **40**, 145005. (doi:10.1088/1361-6382/acdb3f).

This article is part of a discussion meeting issue ‘At the interface of asymptotics, conformal methods and analysis in general relativity’.

## 1. Introduction

One of the main open problems in Mathematical General Relativity is that of the nonlinear stability of spacetimes. In 1986, Friedrich provided the first result concerning the global stability of the de Sitter spacetime and a semi-global stability result for the Minkowski spacetime [1,2]. These results are obtained by using the conformal Einstein field equations to reformulate Cauchy problems which are global or semi-global in time into problems which are local in time. This strategy allows us to use results obtained for quasi-linear symmetric hyperbolic systems [3,4] to prove the existence of solutions which are suitably close to known reference spacetimes. More results [5–12] using the *conformal Einstein field equations* show that these equations are a powerful tool for the analysis of the stability of spacetimes. They provide a system of field equations for geometric objects defined on a four-dimensional Lorentzian manifold (M,g), the so-called *unphysical spacetime*, which is conformally related to a spacetime $\left(\tilde{M},\tilde{g}\right)$, the so-called *physical spacetime*, satisfying the Einstein field equations. The conformal Einstein field equations constitute a system of differential conditions on the curvature tensors with respect to the Levi–Civita connection of g and the conformal factor Ξ.

A problem one encounters when discussing the conformal structure of spacetimes by means of these equations is that of the gauge freedom. In the original formulation of the conformal Einstein field equations [13] the gauge is fixed by means of *gauge source functions*. An alternative approach of gauge fixing is by exploiting the properties of a congruence of curves which are invariants of the conformal structure. These curves are known as *conformal geodesics* and they have been originally introduced as a tool for the local analysis of the structure of conformally rescaled spacetimes [14]. Using this gauge allows us to define a *conformal Gaussian gauge system* in which coordinates are propagated along conformal geodesics. To combine this gauge choice with the conformal Einstein field equations, it is necessary to make use of a more general version of the latter, the *extended conformal Einstein field equations*. These equations contain a larger gauge freedom due to the use of a *Weyl connection*. This is a torsion-free connection which provides a transport equation along the conformal geodesics preserving conformally orthonormal frames and the causal nature of their vectors.

One of the advantages of the *conformal Gaussian gauge system* is that it gives an *a priori* knowledge of the structure of the conformal boundary of the spacetime. This aspect is used to obtain an alternative proof of the semi-global nonlinear stability of the Minkowski spacetime and of the global nonlinear stability of the de Sitter spacetime by Lübbe and Valiente Kroon [8]. In [15] the results obtained in [1,8] are generalized to de Sitter-like spacetimes with compact spatial sections of negative scalar curvature. The existence and stability result follows from explicit calculations and the requirement that the data are close to de Sitter-like data. The success of this approach in the analysis of the global properties of asymptotically simple spacetimes leads to the question of whether a similar strategy can be used to study the evolution of black hole spacetimes. A first step in this direction is made in [16] where certain aspects of the conformal structure of the sub-extremal Schwarzschild–de Sitter spacetime are analysed in order to adapt techniques from the asymptotically simple setting to the black hole case. More precisely, since this solution can be studied by means of the extended conformal Einstein field equations—see [17]. These equations are used to obtain a result concerning the evolution of the region of this spacetime which is bounded by the Cosmological horizon known as the *Cosmological region*. In particular, in analogy to the de Sitter-like case, the Cosmological region has an asymptotic region admitting a smooth conformal extension with a space-like conformal boundary and there exists a conformal representation in which the induced 3-metric on the conformal boundary I is homogeneous. Thus, it is possible to integrate the extended conformal field equations along single conformal geodesics—see [18,19].

In this review article, the discussion of the construction of a conformal Gaussian gauge system leading to a hyperbolic reduction of the conformal Einstein field equations in the de Sitter-like case [15] and the sub-extremal Schwarzschild–de Sitter case [16] is revisited and presented in a coherent and contiguous way.

### (a) Notations and conventions

The signature convention for Lorentzian spacetime metrics will be (−,+,+,+). In this article, the abstract index notation is used. Accordingly, the lowercase Latin indices {a, b, c, …} will denote spacetime abstract tensor indices and ${\{}_{a}{,}_{b}{,}_{c},\ldots \}$ will be used as spacetime frame indices taking the values 0,…,3. In this way, given a basis $\left\{e_{a}\right\}$ a generic tensor is denoted by $T_{ab}$ while its components in the given basis are denoted by $T_{ab}\equiv T_{ab}e_{a}{}^{a}e_{b}{}^{b}$. The Greek indices ${}_{\mu },{}_{\nu },\ldots$ denote spacetime coordinate indices while the indices ${}_{\alpha },{}_{\beta },\ldots$ denote spatial coordinate indices. An index-free notation is used where convenient. Given a 1-form ω and a vector v, the action of ω on v is denoted by ⟨ω,v⟩. The *musical isomorphisms* ${}^{♯}$ and ${}^{♭}$ are used to denote the contravariant version $\omega^{♯}$ of ω and the covariant version $v^{♭}$ of v with respect to a given Lorentzian metric g. This notation can be extended to tensors of higher rank.

The conventions for the curvature tensors are fixed by the relation

$$(\nabla_{a}\nabla_{b}-\nabla_{b}\nabla_{a})v^{c}=R^{c}{}_{dab}v^{d}.$$

## 2. Tools of conformal geometry

The purpose of this section is to provide a brief summary of the technical tools of conformal geometry that will be used in the analysis of the evolution of the spacetimes under consideration.

Let $\left(\tilde{M},\tilde{g}\right)$ be a vacuum spacetime satisfying the Einstein field equations with positive Cosmological constant

2.1 $${\tilde{R}}_{ab}=\lambda {\tilde{g}}_{ab}$$

and let g denote an unphysical Lorentzian metric conformally related to $\tilde{g}$ via the relation

$$g=\Xi^{2}\tilde{g}$$

with Ξ a suitable conformal factor. The Levi–Civita connections of the metrics g and $\tilde{g}$ are denoted by ∇ and $\tilde{\nabla }$, respectively. The set of points for which Ξ=0 is called the *conformal boundary*.

### (a) Weyl connections

A Weyl connection is a torsion-free connection $\hat{\nabla }$ such that

$${\hat{\nabla }}_{a}g_{bc}=-2f_{a}g_{bc}.$$

It follows from the above that the connections $\nabla_{a}$ and ${\hat{\nabla }}_{a}$ are related to each other by

2.2 $${\hat{\nabla }}_{a}v^{b}-\nabla_{a}v^{b}=S_{ac}{}^{bd}f_{d}v^{c},\;S_{ac}{}^{bd}\equiv \delta_{a}{}^{b}\delta_{c}{}^{d}+\delta_{a}{}^{d}\delta_{c}{}^{b}-g_{ac}g^{bd},$$

where $f_{a}$ is a fixed smooth covector and $v^{a}$ is an arbitrary vector. Given that

$$\nabla_{a}v^{b}-{\tilde{\nabla }}_{a}v^{b}=S_{ac}{}^{bd}(\Xi^{-1}\nabla_{a}\Xi )v^{c},$$

one has that

$${\hat{\nabla }}_{a}v^{b}-{\tilde{\nabla }}_{a}v^{b}=S_{ac}{}^{bd}\beta_{d}v^{c},\;\beta_{d}\equiv f_{d}+\Xi^{-1}\nabla_{d}\Xi .$$

In the following, it will be convenient to define

2.3 $$d_{a}\equiv \Xi f_{a}+\nabla_{a}\Xi .$$

### (b) The frame version of the extended conformal Einstein field equations

The *extended conformal Einstein field equations* constitute a conformal representation of the vacuum Einstein field equations written in terms of *Weyl connections*—see [20]. These equations are formally regular at the conformal boundary. Moreover, a solution to the extended conformal equations implies, in turn, a solution to the vacuum Einstein field equations away from the conformal boundary.

Let $\left\{e_{a}\right\}$, a=0,…,3 denote a g-orthogonal frame with associated coframe $\left\{\omega^{a}\right\}$. Thus, one has that

$$g(e_{a},e_{b})=\eta_{ab}\;\mathrm{and}\;\langle \omega^{a},e_{b}\rangle =\delta_{b}{}^{a}.$$

The frame formulation of the *extended conformal Einstein field equations* is obtained by defining the following *zero-quantities*:

2.4*a* $$\begin{array}{rl} & \hat{\Sigma }{}_{a}{}^{c}{}_{b}e_{c}\equiv [e_{a},e_{b}]-(\hat{\Gamma }{}_{a}{}^{c}{}_{b}-\hat{\Gamma }{}_{b}{}^{c}{}_{a})e_{c}, \end{array}$$

2.4*b* $$\begin{array}{rl} & \hat{\Xi }{}^{c}{}_{dab}\equiv \hat{R}{}^{c}{}_{dab}-\hat{\rho }{}^{c}{}_{dab}, \end{array}$$

2.4*c* $$\begin{array}{rl} & \hat{\Delta }{}_{cdb}\equiv {\hat{\nabla }}_{c}\hat{L}{}_{db}-{\hat{\nabla }}_{d}\hat{L}{}_{cb}-d_{a}d{}^{a}{}_{bcd} \end{array}$$

2.4*d* $$\begin{array}{rl} \;\mathrm{and}\;\; & \Lambda {}_{bcd}\equiv {\hat{\nabla }}_{a}d^{a}{}_{bcd}-f_{a}d{}^{a}{}_{bcd}, \end{array}$$

where the components of the *geometric curvature* $\hat{R}{}^{c}{}_{dab}$ and the *algebraic curvature* $\hat{\rho }{}^{c}{}_{dab}$ are given, respectively, by

$$\hat{R}{}^{c}{}_{dab}\equiv \partial_{a}(\hat{\Gamma }{}_{b}{}^{c}{}_{d})-\partial_{b}(\hat{\Gamma }{}_{a}{}^{c}{}_{d})+\hat{\Gamma }{}_{f}{}^{c}{}_{d}(\hat{\Gamma }{}_{b}{}^{f}{}_{a}-\hat{\Gamma }{}_{a}{}^{f}{}_{b})+\hat{\Gamma }{}_{b}{}^{f}{}_{d}\hat{\Gamma }{}_{a}{}^{c}{}_{f}-\hat{\Gamma }{}_{a}{}^{f}{}_{d}\hat{\Gamma }{}_{b}{}^{c}{}_{f}$$

and

$$\hat{\rho }{}^{c}{}_{dab}\equiv \Xi \hat{d}{}^{c}{}_{dab}+2S{}_{d[a}{}^{ce}\hat{L}{}_{b]e}.$$

In terms of the zero-quantities (2.4*a*–d), the *extended conformal Einstein field equations* are given by the conditions

2.5 $$\hat{\Sigma }{}_{a}{}^{c}{}_{b}e_{c}=0,\;\hat{\Xi }{}^{c}{}_{dab}=0,\;\hat{\Delta }{}_{cdb}=0\;\mathrm{and}\;\Lambda {}_{bcd}=0.$$

In the above equations, the fields Ξ and $d_{a}$ are regarded as *conformal gauge fields* which are determined by gauge conditions. These conditions will be determined through conformal geodesics—see §2d. In order to account for this, we write down the gauge equations as

2.6*a* $$\begin{array}{rl} & \delta_{a}\equiv d_{a}-\Xi f_{a}-{\hat{\nabla }}_{a}\Xi , \end{array}$$

2.6*b* $$\begin{array}{rl} & \gamma_{ab}\equiv {\hat{L}}_{ab}-{\hat{\nabla }}_{a}(\Xi^{-1}d_{b})-\frac{1}{2}\Xi^{-1}S_{ab}{}^{cd}d_{c}d_{d}+\frac{1}{6}\lambda \Xi^{-2}\eta_{ab} \end{array}$$

2.6*c* $$\begin{array}{rl} \;\mathrm{and}\; & \varsigma_{ab}\equiv {\hat{L}}_{\left[ab\right]}-{\hat{\nabla }}_{[a}f_{b]}. \end{array}$$

Then, the conditions

2.7 $$\delta_{a}=0,\;\gamma_{ab}=0\;\mathrm{and}\;\varsigma_{ab}=0,$$

will be called the *supplementary conditions*. They play a role in relating the Einstein field equations to the extended conformal Einstein field equations and also in the propagation of the constraints.

The correspondence between the Einstein field equations and the extended conformal Einstein field equations is given by the following—see proposition 8.3 in [11]:

**Proposition 2.1.**

*Let*

$$\left(e_{a},{\hat{\Gamma }}_{a}{}^{b}{}_{c},{\hat{L}}_{ab},d^{a}{}_{bcd}\right)$$

*denote a solution to the extended conformal Einstein field equations* (2.5) *for some choice of the conformal gauge fields* $\left(\Xi ,d_{a}\right)$ *satisfying the supplementary conditions (2.7). Furthermore, suppose that*

$$\Xi \neq 0\;\mathrm{and}\;\det(\eta^{ab}e_{a}⊗e_{b})\neq 0$$

*on an open subset* U. *Then the metric*

$$\tilde{g}=\Xi^{-2}\eta_{ab}\omega^{a}⊗\omega^{b}$$

*is a solution to the Einstein field equations (2.1) on* U.

### (c) The conformal Einstein constraint equations

The *conformal Einstein constraint equations* are intrinsic equations implied by the standard conformal Einstein field equations on a space-like hypersurface.

Let S denote a space-like hypersurface in an unphysical spacetime (M,g) and let $\left\{e_{a}\right\}$ denote a g-orthonormal frame adapted to S. Now, let h be the *intrinsic metric* of S with associated Levi–Civita connection D. Since $D_{i}=e{}_{i}{}^{i}D_{i}$ is the directional covariant derivative acting on hypersurface-defined objects. The conformal constraint equations in the vacuum case are given by—see [11]:

2.8*a* $$\begin{array}{rl} & D_{i}D_{j}\Omega =\Sigma \chi_{ij}-\Omega L_{ij}+sh_{ij}, \end{array}$$

2.8*b* $$\begin{array}{rl} & D_{i}\Sigma ={\chi_{i}}^{k}D_{k}\Omega -\Omega L_{i}, \end{array}$$

2.8*c* $$\begin{array}{rl} & D_{i}s=L_{i}\Sigma -L_{ik}D^{k}\Omega , \end{array}$$

2.8*d* $$\begin{array}{rl} & D_{i}L_{jk}-D_{j}L_{ik}=\Sigma d_{kij}+D^{l}\Omega d_{lkij}-(\chi_{ik}L_{j}-\chi_{jk}L_{i}), \end{array}$$

2.8*e* $$\begin{array}{rl} & D_{i}L_{j}-D_{j}L_{i}=D^{l}\Omega d_{lij}+{\chi_{i}}^{k}L_{jk}-{\chi_{j}}^{k}L_{ik}, \end{array}$$

2.8*f* $$\begin{array}{rl} & D^{k}d_{kij}=-({\chi^{k}}_{i}d_{jk}-{\chi^{k}}_{j}d_{ik}), \end{array}$$

2.8*g* $$\begin{array}{rl} & D^{i}d_{ij}=\chi^{ik}d_{ijk}, \end{array}$$

2.8*h* $$\begin{array}{rl} & \lambda =6\Omega s+3\Sigma^{2}-3D_{k}\Omega D^{k}\Omega , \end{array}$$

2.8*i* $$\begin{array}{rl} & D_{j}\chi_{ki}-D_{k}\chi_{ji}=\Omega d_{ijk}+h_{ij}L_{k}-h_{ik}L_{j} \end{array}$$

2.8*j* $$\begin{array}{rl} \;\mathrm{and}\; & l_{ij}=\Omega d_{ij}+L_{ij}-\chi \left(\chi_{ij}-\frac{1}{4}\chi h_{ij}\right)+\chi_{ki}{\chi_{j}}^{k}-\frac{1}{4}\chi_{kl}\chi^{kl}h_{ij}, \end{array}$$

with the understanding that

$$h_{ij}\equiv g_{ij}=\delta_{ij}$$

and where

$$L_{i}\equiv L_{0i},\;d_{ij}\equiv d_{0i0j}\;\mathrm{and}\;d_{ijk}\equiv d_{i0jk}.$$

Moreover, Ω denotes the restriction of the spacetime conformal factor Ξ to S and Σ is the normal component of the gradient of Ξ. The field $l_{ij}$ denotes the components of the Schouten tensor of the induced metric $h_{ij}$ on S. The fields $d_{ij}$ and $d_{ijk}$ correspond, respectively, to the electric and magnetic parts of the rescaled Weyl tensor. The scalar s denotes the *Friedrich scalar* defined as

$$s\equiv \frac{1}{4}\nabla_{a}\nabla^{a}\Xi +\frac{1}{24}R\Xi ,$$

with R the Ricci scalar of the metric g. Finally, $L_{ij}$ denotes the spatial components of the Schouten tensor of g.

### (d) Conformal geodesics

The extended conformal Einstein field equations, being expressed in terms of a Weyl connection, contain a larger gauge freedom than the standard conformal Einstein field equations. In this case, the gauge used to analyse the evolution of the spacetimes under consideration is based on the properties of *conformal geodesics*.

#### (i) Basic definitions

A ***conformal geodesic*** on a spacetime $\left(\tilde{M},\tilde{g}\right)$ is a pair (x(τ),β(τ)) consisting of a curve x(τ) and a covector β(τ) along x(τ) satisfying the equations

2.9*a* $${\tilde{\nabla }}_{\dot{x}}\dot{x}=-2\langle \beta ,\dot{x}\rangle \dot{x}+\tilde{g}(\dot{x},\dot{x})\beta^{♯}$$

and

2.9*b* $${\tilde{\nabla }}_{\dot{x}}\beta =\langle \beta ,\dot{x}\rangle \beta -\frac{1}{2}{\tilde{g}}^{♯}(\beta ,\beta ){\dot{x}}^{♭}+\tilde{L}(\dot{x},\cdot ),$$

where $\tilde{L}$ denotes the *Schouten tensor* of the Levi–Civita connection $\tilde{\nabla }$. A vector v is said to be *Weyl propagated* if along x(τ) it satisfies the equation

2.10 $${\tilde{\nabla }}_{\dot{x}}v=-\langle \beta ,v\rangle \dot{x}-\langle \beta ,\dot{x}\rangle v+\tilde{g}(v,\dot{x})\beta^{♯}.$$

The *conformal geodesic equations* (2.9*a*) and (2.9*b*) are conformally invariant, in the sense that if a solution is obtained with one representative of the conformal class of metrics, then a simple transformation of the solution exists to map it into a solution with respect to another representative. Hence, these curves are coming solely from the *conformal structure*.

A congruence of conformal geodesics can be used to single out a metric g out of the equivalence class of conformally related metrics $\left[\tilde{g}\right]$. This is due to the following property:

**Proposition 2.2.**

*Let* $\left(\tilde{M},\tilde{g}\right)$ *denote a vacuum spacetime with positive Cosmological constant. Suppose that* (x(τ),β(τ)) *is a solution to the conformal geodesic equations (2.9a*,b) *and that* $\left\{e_{a}\right\}$ *is a* g-*orthonormal frame propagated along the curve according to equation (2.10). If* Θ *satisfies*

2.11 $$g(\dot{x},\dot{x})=-1\;\mathrm{and}\;g=\Theta^{2}\tilde{g},$$

*then one has that*

2.12 $$\Theta (\tau )=\Theta_{⋆}+{\dot{\Theta }}_{⋆}(\tau -\tau_{⋆})+\frac{1}{2}{\ddot{\Theta }}_{⋆}{\left(\tau -\tau_{⋆}\right)}^{2},$$

*where the coefficients*

$$\Theta_{⋆}\equiv \Theta (\tau_{⋆}),\;{\dot{\Theta }}_{⋆}\equiv \dot{\Theta }(\tau_{⋆})\;\mathrm{and}\;{\ddot{\Theta }}_{⋆}\equiv {\ddot{\Theta }}_{⋆}(\tau_{⋆})$$

*are constant along the conformal geodesic and are subject to the constraints*

$${\dot{\Theta }}_{⋆}=\langle \beta_{⋆},{\dot{x}}_{⋆}\rangle \Theta_{⋆}\;\mathrm{and}\;\Theta_{⋆}{\ddot{\Theta }}_{⋆}=\frac{1}{2}{\tilde{g}}^{♯}(\beta_{⋆},\beta_{⋆})+\frac{1}{6}\lambda .$$

A proof of this result can be found in [11].

Thus, if a spacetime can be covered by a non-intersecting congruence of conformal geodesics, then the location of the conformal boundary is known *a priori* in terms of data at a fiduciary initial hypersurface $S_{⋆}$.

#### (ii) The $\tilde{g}$-adapted conformal geodesic equations

As a consequence of the normalization condition (2.11), the parameter τ is the g-proper time of the curve x(τ). In some computations it is more convenient to consider a parametrization in terms of a $\tilde{g}$-proper time $\tilde{\tau }$ of the curve $\tilde{x}(\tilde{\tau })$ as it allows us to work directly with the physical metric. To this end, consider the parameter transformation $\tilde{\tau }=\tilde{\tau }(\tau )$ given by

2.13 $$\frac{d\tau }{d\tilde{\tau }}=\Theta ,\;\text{so that }\tilde{\tau }={\tilde{\tau }}_{⋆}+\int_{\tau_{⋆}}^{\tau }\frac{\text{ds}}{\Theta (\text{s})},$$

with inverse $\tau =\tau (\tilde{\tau })$. Now, consider

2.14 $${\tilde{x}}^{\prime }=\Theta \dot{x},\;\beta =\tilde{\beta }+\varpi {\dot{x}}^{♭}\;\mathrm{and}\;\varpi \equiv \frac{\left\langle \beta ,\dot{x}\right\rangle }{\tilde{g}(\dot{x},\dot{x})},$$

in equations (2.9*a*,b) so that one obtains the following $\tilde{g}$-**adapted equations for the conformal geodesics**:

2.15*a* $${\tilde{\nabla }}_{{\tilde{x}}^{\prime }}{\tilde{x}}^{\prime }={\tilde{\beta }}^{♯},$$

and

2.15*b* $${\tilde{\nabla }}_{{\tilde{x}}^{\prime }}\tilde{\beta }={\tilde{\beta }}^{2}{\tilde{x}}^{\prime }{}^{♭}+\tilde{L}({\tilde{x}}^{\prime },\cdot )-\tilde{L}({\tilde{x}}^{\prime },{\tilde{x}}^{\prime }){\tilde{x}}^{\prime ♭},$$

with ${\tilde{\beta }}^{2}\equiv {\tilde{g}}^{♯}(\tilde{\beta },\tilde{\beta })$. For a vacuum spacetime with Cosmological constant one has that

$$\tilde{L}=\frac{1}{6}\lambda \tilde{g}.$$

### (e) A conformal Gaussian gauge system

The use of the extended conformal Einstein field equations and a gauge adapted to a congruence of conformal geodesics allows the construction of *conformal Gaussian gauge systems*. To construct a *conformal Gaussian gauge system*, one considers a region U of the spacetime (M,g) which is covered by a non-intersecting congruence of conformal geodesics (x(τ),β(τ)) and proceeds as follows:

— The property of the congruence of conformal geodesics stated by proposition 2.2 allows one to single out a *canonical representative* g of the conformal class $\left[\tilde{g}\right]$ with an explicitly known conformal factor Θ as given by the formula (2.12).
— Let $\left\{e_{a}\right\}$ denote a g-orthonormal frame which is Weyl propagated along the conformal geodesics. To every congruence of conformal geodesics one can associate a Weyl connection ${\hat{\nabla }}_{a}$ by setting $f_{a}=\beta_{a}$. It follows that for this connection one has
  $${\hat{\Gamma }}_{0}{}^{a}{}_{b}=0,\;f_{0}=0\;\mathrm{and}\;{\hat{L}}_{0a}=0.$$
  This gauge choice is supplemented by choosing the parameter τ of the conformal geodesics as the time coordinate so that
  $$e_{0}=\partial_{\tau }.$$
— Since the initial data for the congruence of conformal geodesics is prescribed on a fiduciary space-like hypersurface $S_{⋆}$. On $S_{⋆}$ one can choose some local coordinates $\underline{x}=(x^{\alpha })$. These coordinates can be extended off $S_{⋆}$ by requiring them to remain constant along the conformal geodesic which intersects $S_{⋆}$ at the point p with coordinates $\underline{x}$. The spacetime coordinates $\bar{x}=(\tau ,x^{\alpha })$ obtained in this way are known as *conformal Gaussian coordinates*.

The collection of conformal factor Θ, Weyl propagated frame $\left\{e_{a}\right\}$ and coordinates $\left(\tau ,x^{\alpha }\right)$ is known as a *conformal Gaussian gauge system*.

One of the advantages of this procedure is that one has an *a priori* knowledge of the location of the conformal boundary. This is in contrast with other conformal gauges in which the conformal factor is an unknown. The use of a conformal Gaussian gauge system leads to a particularly simple system of conformal evolution equations. The evolution of all the geometric unknowns is either fixed by the gauge or given by transport equations along the congruence of conformal geodesics. Moreover, whether the standard Gaussian gauge, adapted to metric geodesics, is known to develop caustics. However, by looking at the geodesic deviation equation for the conformal geodesics, extra terms appear involving the 1-form that counter the contribution from the curvature. Thus, these curves are less likely to develop caustics. Besides, since in [18], Friedrich shows that there exists a congruence of conformal geodesics in the Schwarzschild spacetime that yields a semi-global frame that is regular up to and beyond future null infinity. The use of a gauge adapted to these curves is a more convenient choice.

## 3. Standard results for symmetric hyperbolic systems

The nature of the conformal Einstein field equations requires the *hyperbolic reduction* of the conformal evolution equations to discuss the existence and asymptotic properties of their solutions. In this case, the existence of a conformal Gaussian gauge system allows a hyperbolic reduction of the conformal evolution equations as a quasi-linear symmetric hyperbolic system. The purpose of this section is to provide a brief summary of the known technical results for quasi-linear symmetric hyperbolic systems that will be used in the analysis of the evolution of the spacetimes under consideration.

### (a) Kato’s theorem on symmetric hyperbolic systems

Kato’s theory is concerned with symmetric hyperbolic systems in which the unknown u is regarded as a P-valued function over $R^{m}$ where P is a Hilbert space. The Hilbert space can be real or complex and infinite dimensional. In this article, we are interested in the case where $P=R^{N}$ and m=3. In this case, the symmetric hyperbolic system becomes a *standard* partial differential equation.

Given a N-dimensional symmetric hyperbolic quasi-linear systems of the form

3.1 $${\text{A}}^{0}(t,\underline{x},\text{u})\partial_{t}\text{u}+{\text{A}}^{\alpha }(t,\underline{x},\text{u})\partial_{\alpha }\text{u}=\text{B}(t,\underline{x},\text{u}),$$

for 0≤t≤T, $\underline{x}\in R^{3}$, α=1,2,3, and initial conditions

3.2 $$\text{u}(0,x)={\text{u}}_{⋆}(\underline{x}).$$

In Kato’s theory, the coefficients $A^{0}(t,\underline{x},u)$ and $A^{\alpha }(t,\underline{x},u)$ are nonlinear operators depending on t sending $R^{N}$-valued functions over $R^{3}$ into (N×N)-matrix valued functions on $R^{3}$. Similarly, $B(t,\underline{x},u)$ is a nonlinear operator depending on t sending $R^{N}$-valued functions on $R^{3}$ into $R^{N}$-valued functions on $R^{3}$.

Consider $H^{s}(R^{3},R^{N})$, the space of $\left(R^{N}\right)$-vector valued functions over $R^{3}$ such that their entries have finite Sobolev norm of order s. Let R be a bounded open subset of $H^{s}(R^{3},R^{N})$. Writing

$$\begin{array}{rl} & {\text{A}}^{\mu }(t,\underline{x},\text{u})=(a{}^{\mu }{}_{ij}(t,\underline{x},\text{u}))\\ & \;\mathrm{and}\;\\ & \text{B}(t,\underline{x},\text{u})=(b_{i}(t,\underline{x},\text{u})),\;i,j=1,\ldots N,\;\mu =0,\ldots ,3, \end{array}$$

one has that for fixed t and u∈R

$$a{}^{\mu }{}_{ij}(t,\underline{x},\text{u}):R^{m}\to R$$

and

$$b_{i}(t,\underline{x},\text{u}):R^{m}\to R.$$

Now, let $C_{0}^{\infty }(R^{3},R)$ denote the sets of smooth functions of compact support from $R^{3}$ to R. Given any non-zero $\phi \in C_{0}^{\infty }(R^{3},R)$ not identically zero, then u belongs to the *uniformly local Sobolev spaces* $H_{ul}^{s}$ if and only if

$$\underset{\underline{x}\in R^{3}}{\sup}∥\phi_{x}\text{u}{∥}_{s}<\infty ,\;\phi_{x}(\underline{y})\equiv \phi (\underline{y}-\underline{x}).$$

In the following, for fixed t and u∈R, the coefficients $a_{ij}^{\mu }(t,\underline{x},u(\underline{x}))$ are functions from R to $H_{ul}^{s}(R^{3},R)$ whereas $b_{i}(t,\underline{x},u(\underline{x}))$ is a function from R to $H^{s}(R^{3},R)$. In Kato’s terminology this is equivalent to requiring that $A^{\mu }$ is a function from R to $H_{ul}^{s}(R^{3},B(P))$ and B from R to $H^{s}(R^{3},P)$. Accordingly, one has the following reformulation of Theorem II in [4]:

**Theorem 3.1.**

*Let* s *be a positive integer such that* s>3/2+1=5/2. *Let* $A^{\mu }(t,\underline{x},v(\underline{x}))$, $B(t,\underline{x},v(\underline{x}))$ *and* v∈R *as above with* 0≤t≤T. *Assume that the following conditions hold*:

(i) *The components* $a_{ij}^{\mu }(t,\underline{x},v(\underline{x}))$ (*respectively*, $b_{i}(t,\underline{x},v(\underline{x}))$) *are bounded in the* $H_{ul}^{s}$-*norm* (*respectively* $H^{s}$-*norm*) *for* v∈R, *uniformly in* t.
(ii) *For each* t, *the map* $v(\underline{x})\mapsto A^{\alpha }(t,\underline{x},v(\underline{x}))$ *is uniformly Lipschitz continuous on* R *from the* $H^{0}$-*norm to the* $H_{ul}^{0}$-*norm, uniformly in* t. *Similarly, the map* $v(\underline{x})\mapsto B(t,\underline{x},v(\underline{x}))$ *is Lipschitz continuous from the* $H^{0}$-*norm to the* $H^{0}$-*norm, again uniformly in* t.
(iii) *The map* $v(\underline{x})\mapsto A^{0}(t,\underline{x},v(\underline{x}))$ *is Lipschitz continuous on* R *from the* $H^{s-1}$-*norm to the* $H_{ul}^{s-1}$-*norm, uniformly in* t.
(iv) *The maps* $t\mapsto A^{\alpha }(t,\underline{x},v(\underline{x}))$ *are continuous in the* $H_{ul}^{0}$-*norm for each* v∈R. *Similarly, the map* $t\mapsto B(t,\underline{x},v(\underline{x}))$ *is continuous in the* $H^{0}$-*norm for each* v∈R.
(v) *The map* $t\mapsto A^{0}(t,\underline{x},v(\underline{x}))$ *is Lipschitz-continuous on* [0,T] *in the* $H_{ul}^{s-1}$-*norm, uniformly for* v∈R.
(vi) *For each* v∈R *the matrix-valued functions* $A^{\mu }(t,\underline{x},v(\underline{x}))$ *are symmetric for each* $(t,\underline{x})\in [0,T]\times R^{m}$.
(vii) *The matrix* $A^{0}(t,\underline{x},v(\underline{x}))$ *is positive definite with eigenvalues larger that, say, 1 for each* $\left(t,\underline{x}\right)$ *and each* v∈R.
(viii) $u_{⋆}\in R$.

*Then there is a unique solution* u *to* (3.1) *and* (3.2) *defined on* $\left[0,T^{\prime }\right]$ *where* $0<T^{\prime }\leq T$ *such that*

$$\text{u}\in C[0,T^{\prime };R]\cup C^{1}[0,T^{\prime };H^{s-1}(R^{3},R^{N})],$$

*where* $T^{\prime }$ *can be chosen common to all initial conditions* $u_{⋆}$ *in a suitably small condition of a given point in* R.

Since the conditions of the above theorem are hard to verify, Kato provides sufficient conditions for these requirements to be satisfied:

**Theorem 3.2.**

*Suppose that* s>3/2+1=5/2. *Let* Ω *be the subset of* $R^{3}\times R^{N}$ *consisting of pairs* $\left(\underline{x},\underline{v}\right)$ *such that*

$$|v-v_{⋆}(x)|<\omega ,\;\underline{x}\in R^{3}$$

*where* ω>0 *and* $v_{⋆}\in H^{s}(R^{3},R^{N})\subset C^{1}(R^{3},R^{N})$ *are fixed. Let*

$${\text{A}}^{\mu }:[0,T]\times \Omega ⟶B(R^{N})$$

*and*

$$\text{B}:[0,T]\times \Omega ⟶R^{N},$$

*where* $B(R^{N})$ *denotes the set of* (N×N)-*matrix valued functions over* $R^{3}$ *with the properties*

(a) $A^{\alpha }\in C[0,T;C_{b}^{s}(\Omega ,B(R^{N}))]$,
(b) $A^{0}\in Lip[0,T;C_{b}^{s-1}(\Omega ,B(R^{N}))]$,
(c) $B\in C[0,T;C_{b}^{s+1}(\Omega ,R^{N})]$,
(d) $B_{⋆}\in L^{\infty }[0,T;H^{s}(R^{3},R^{N})]\cap C[0,T;H^{0}(R^{3},R^{N})]$,

*where* $B_{⋆}(t,\underline{x})\equiv B(t,\underline{x},v_{⋆}(\underline{x}))$ *and the sets* $C_{b}^{r}(\Omega ,B(R^{N}))$ and $C_{b}^{r}(\Omega ,R^{N})$ *denote the spaces of functions having derivatives up to the* r*th order which are continuous and bounded in the supremum norm. Then conditions (i)–(v) in theorem 3.1 are satisfied by* $A^{\mu }$, B *provided that* R *is chosen as a ball in* $H^{s}(R^{3},R^{N})$ *with* $v_{⋆}$ *as centre and a sufficiently small radius* $R_{⋆}$. *In addition, (ix) is satisfied if (a) is assumed to hold with* s *replaced by* s+1.

### (b) Existence and stability result for symmetric hyperbolic systems on manifolds with compact spatial sections

The result contained in theorem 3.1 can be extended to Cauchy problems for symmetric hyperbolic system with data prescribed on compact three-dimensional manifolds.

Given a compact three-dimensional manifold S, there exists a finite cover consisting of open sets $R_{1},\ldots ,R_{M}\subset S$ such that $\cup_{i=1}^{M}R{}_{i}=S$. On each of the open sets $R{}_{i}$ it is possible to introduce coordinates $\underline{x}{}_{i}\equiv (x{}^{\alpha }{}_{i})$ which allow one to identify $R{}_{i}$ with open subsets $B_{i}\subset R^{3}$. As S is assumed to be a smooth manifold, the coordinate patches can be chosen so that the change of coordinates on intersecting sets is smooth. The initial data $u{}_{⋆}:S\to R^{N}$ are a smooth function on S and can be restricted to a particular open set $R{}_{i}$. The restriction $u{}_{i⋆}$, in local coordinates $x_{i}$, can be regarded as a function $u_{i⋆}:B_{i}\to R^{N}$. Now, assuming that $R\subset R^{3}$ is bounded with smooth boundary ∂R, it is possible to extend $u_{i⋆}$ to a function $Eu_{i⋆}:R^{3}\to R^{N}$ by means of the following proposition:

**Proposition 3.3.**

*Let* $R\subset R^{3}$ *be a bounded region with smooth boundary* ∂R. *Then there exists a linear operator*

$$E:H^{m}(R,R^{N})⟶H^{m}(R^{3},R^{N})$$

*such that for each* $u\in H^{m}(R,R^{N})$:

(i) Eu=u *almost everywhere in* R;
(ii) Eu *has support in an open bounded set* $R^{\prime }$ *with* $R\subset R^{\prime }$;
(iii) *There exists a constant* C *depending only on* U *and* R *such that*
  $$||Eu|{|}_{R^{3},m}\leq C||u|{|}_{R,m}.$$

*The* $R^{N}$-*valued function* Eu *is an* *extension* *of* u *to* $R^{3}$.

— see [11,21].

Using these extensions, it is possible to define the Sobolev norm

$$||{\text{u}}_{⋆}|{|}_{S,m}\equiv \sum_{i=1}^{M}||\text{u}{}_{i⋆}|{|}_{R^{3},m}.$$

Now, for each of the $Eu_{i⋆}$ one can formulate an initial value problem of the form

$${\bar{\text{A}}}^{0}(t,\underline{x},\text{u})\partial_{t}\text{u}+{\bar{\text{A}}}^{\alpha }(t,\underline{x},\text{u})\partial_{\alpha }\text{u}=B(t,\underline{x},\text{u})$$

and

$$\text{u}(0,\underline{x})=E{\text{u}}_{i⋆}(\underline{x})\in H{}^{m}(S,R{}^{N})\;\mathrm{for}\;m\geq 4.$$

If this system satisfies the conditions of theorem 3.1 the theory implies existence, uniqueness and stability. However, this theorem only applies to settings in which the spatial sections are diffeomorphic to $R^{3}$. One makes use of standard results on causality theory implying that

$$D^{+}(R_{i})\cap I^{+}(S\setminus R_{i})=\emptyset ,$$

where $D^{+}(R_{i})$ denotes the causal future of $R_{i}$—see e.g. [11], theorem 14.1. Accordingly, the value of u on $D_{i}\equiv D^{+}(R_{i})$ is determined only by the data on $R_{i}$. Then the solution on $D_{i}$ is independent of the particular extension $Eu_{i⋆}$ being used. Hence, one can speak of a solution $u_{i}$ on a domain $D_{i}\subset [0,t_{i}]\times R_{i}$. Since the manifold is smooth and as a consequence of uniqueness, it follows that given two solutions $u_{i}$ and $u_{j}$ defined, respectively, on intersecting domains $D_{i}$ and $D_{j}$ they must coincide on $D_{i}⋂D_{j}$. Proceeding in the same manner over the whole finite cover of S and since the compactness of S ensures the existence of a minimum non-zero existence time for the whole of the domains $D_{i}$, then there is a unique solution u on [0,T]×S with $T=\min_{i=1,\ldots ,M}\{t_{i}\}$ which is constructed by patching together the localized solutions $u_{1},\ldots ,u_{M}$ defined, respectively, on the domains $D_{i},\ldots ,D_{M}$. This result is summarized by the following theorem [11]:

**Theorem 3.4.**

*Consider the Cauchy problem for a quasi-linear symmetric hyperbolic system*

$${\text{A}}^{0}(t,\underline{x},\text{u})\partial_{t}\text{u}+{\text{A}}^{\alpha }(t,\underline{x},\text{u})\partial_{\alpha }\text{u}=\text{B}(t,\underline{x},\text{u})$$

*and*

$$\text{u}(0,\underline{x})={\text{u}}_{⋆}(\underline{x})\in H^{m}(S,R^{N})\;\mathrm{for}\;m\geq 4$$

*with data on an orientable compact three-dimensional manifold* S. *If there is* δ>0 *such that* $A^{0}(t,\underline{x},u_{⋆})$ *is positive definite with lower bound* δ *for all* x∈S, *then*:

(i) *There exists* T>0 *and a unique solution* u *to the Cauchy problem defined on* [0,T]×S *such that*
  $$u\in C^{m-2}([0,T]\times S,R^{N}).$$
  *Moreover*, $A^{0}(t,\underline{x},u)$ *is positive definite with lower bound* δ *for all* $(t,\underline{x})\in [0,T]\times S$.
(ii) *There exists* ϵ>0 *such that one common existence time* T *can be chosen for all initial conditions in the open ball* $B_{\epsilon }(u_{⋆})$ *and such that* $B_{\epsilon }(u_{⋆})\subset R$.
(iii) *If the solution* u *with initial data* $u_{⋆}$ *exists on* [0,T] *for some* T>0, *then the solutions to all initial conditions in* $B_{\epsilon }(u_{⋆})$ *exists on* [0,T] *if* ϵ>0 *is sufficiently small*.
(iv) *If* ϵ *and* T *are chosen as in* (ii) *and one has a sequence* $u_{⋆}^{n}\in B_{\epsilon }(u_{⋆})$ *such that*
  $$||u_{⋆}^{n}-u_{⋆}|{|}_{S,m}⟶0,\;\mathrm{as}\;n\to \infty ,$$
  *then for the solutions* $u^{n}(t,\cdot )$ *with* $u^{n}(0,\cdot )\equiv u_{⋆}^{n}$ *with it holds that*
  $$||u^{n}-u|{|}_{S,m}⟶0,\;\mathrm{as}\;n\to \infty$$
  *uniformly in* t∈[0,T].

## 4. de Sitter-like spacetimes

In this section, we discuss the evolution of de Sitter-like spacetimes which can be conformally embedded into a portion of a cylinder whose sections have negative scalar curvature as in [15]. The conformal embedding is realized by means of a conformal factor which depends on the affine parameter of the conformal geodesics.

### (a) Basic properties

A de Sitter-like spacetime $\left(\tilde{M},\overset{˚}{\tilde{g}}\right)$ is a solution to the vacuum Einstein field equations with positive Cosmological constant (2.1) given by $\tilde{M}=R\times S$ and

4.1 $$\overset{˚}{\tilde{g}}=-\text{d}t⊗\text{d}t+\sinh^{2}t\;\overset{˚}{\gamma },$$

where $\overset{˚}{\gamma }$ is a positive definite Riemannian metric over a compact manifold S with constant negative curvature. The Riemann curvature tensor $r^{i}{}_{jkl}[\overset{˚}{\gamma }]$ of $\overset{˚}{\gamma }$ is given by

$$r_{ijkl}[\overset{˚}{\gamma }]={\overset{˚}{\gamma }}_{il}{\overset{˚}{\gamma }}_{jk}-{\overset{˚}{\gamma }}_{ik}{\overset{˚}{\gamma }}_{jl}.$$

In particular, by setting λ=3,^1^ it follows from the above expressions that

$$r[\overset{˚}{\gamma }]=-6.$$

Moreover, since

$$\tilde{R}=12,$$

it follows that

4.2 $${\tilde{L}}_{ab}=\frac{1}{2}{\tilde{g}}_{ab}.$$

A spacetime of the form given by $\left(\tilde{M},\overset{˚}{\tilde{g}}\right)$ will be known as a *background solution*.

### (b) Metric geodesics as conformal geodesics

The analysis of the metric geodesics x(s) on $\left(\tilde{M},\overset{˚}{\tilde{g}}\right)$ with $\dot{x}\equiv (\partial x/\partial s)=\alpha \partial_{t}$, where α is a proportionality function, by means of the geodesic equation

$${\tilde{\nabla }}_{\dot{x}}\dot{x}=0$$

and the metric (4.1) shows that α is constant along the integral curves of $\partial_{t}$. Hence, without loss of generality one can set α=1 so that the curves

$$x(t)=(t,{\underline{x}}_{⋆}),\;{\underline{x}}_{⋆}\in S,$$

are non-intersecting time-like $\tilde{g}$-geodesics over $\tilde{M}$. These curves can be recasted as conformal geodesics by means of a reparametrization τ↦t(τ) and a 1-form $\tilde{\beta }$ given by the Ansatz

$$\tilde{\beta }=\alpha (\tau )x^{\prime ♭}=\alpha (t)\;\text{d}t.$$

The resulting pair $\left(x(\tau ),\tilde{\beta }(\tau )\right)$ with

$$x(\tau )=(2\;\text{arctanh}\;\tau ,{\underline{x}}_{⋆}),\;\tilde{\beta }(\tau )=-\frac{2\tau }{1-\tau^{2}}\text{d}\tau \;\mathrm{and}\;\tau \in (-1,1)$$

describes a congruence of non-intersecting time-like conformal geodesics on the background spacetime $\left(\tilde{M},\overset{˚}{\tilde{g}}\right)$.

### (c) The conformal factor associated to the congruence of conformal geodesics

The parameter τ introduced in the previous section is used as a new time coordinate in the metric (4.1) so that

4.3 $$\overset{˚}{\tilde{g}}=\frac{4}{{\left(1-\tau^{2}\right)}^{2}}(-\text{d}\tau ⊗\text{d}\tau +\tau^{2}\overset{˚}{\gamma }).$$

This metric is singular at τ=±1. This line element suggests the introduction of a new unphysical metric $\overset{˚}{g}$ via the relation

$$\overset{˚}{g}=\Theta^{2}\overset{˚}{\tilde{g}},\;\mathrm{with}\;\Theta \equiv \frac{1}{2}\left(1-\tau^{2}\right),$$

so that

4.4 $$\overset{˚}{g}=-\text{d}\tau ⊗\text{d}\tau +\tau^{2}\overset{˚}{\gamma }$$

is well defined for $\tau \in [\tau_{⋆},1]$ with $\tau_{⋆}>0$. The *spatial metric* $\overset{˚}{h}$ is conformally related to $\overset{˚}{\gamma }$ via

$$\overset{˚}{h}\equiv \tau^{2}\overset{˚}{\gamma },$$

with associated Levi–Civita connection to be denoted by $\overset{˚}{D}$, whereas $\overset{˚}{D}$ is the Levi–Civita connection of the metric $\overset{˚}{\gamma }$. The integral curves of the vector field $\partial_{\tau }$ are geodesics of the metric $\overset{˚}{g}$ given by equation (4.4). Moreover, since $\tilde{\beta }$ is a closed 1-form the Weyl connection is, in fact, a Levi–Civita connection which coincides with ∇.

**Figure 1.**
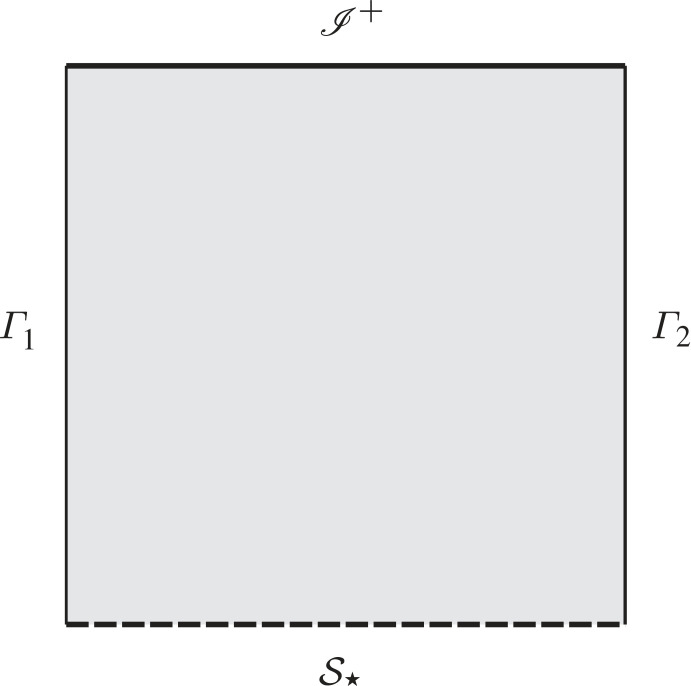
Penrose–Carter diagram of the background solution. The conformal representation discussed in the main text has compact spatial sections of negative scalar curvature. The vertical lines $\Gamma_{1}$ and $\Gamma_{2}$ correspond to axes of symmetry. The solution has a singularity in the past and a space-like future conformal boundary. Hence, in our discussion we only consider future evolution of the initial hypersurface $S_{⋆}$.

A Penrose–Carter diagram of conformal representation of the background solution described by the metric (4.4) is given in figure 1.

### (d) The background spacetime as a solution to the conformal Einstein field equations

The *unphysical spacetime* $\left(M,\overset{˚}{g}\right)$ is recast as a solution to the conformal Einstein field equations. This construction is done using an adapted frame formalism.

#### (i) The frame

Let $\left\{{\overset{˚}{c}}_{i}\right\}$, i=1,2,3, denote a $\overset{˚}{\gamma }$-orthonormal frame over S with associated cobasis $\left\{{\overset{˚}{\alpha }}^{i}\right\}$. Accordingly, one has that

$$\overset{˚}{\gamma }({\overset{˚}{c}}_{i},{\overset{˚}{c}}_{j})=\delta_{ij}\;\mathrm{and}\;\langle {\overset{˚}{\alpha }}^{j},{\overset{˚}{c}}_{i}\rangle =\delta_{i}{}^{j},$$

so that

$$\overset{˚}{\gamma }=\delta_{ij}{\overset{˚}{\alpha }}^{i}⊗{\overset{˚}{\alpha }}^{j}.$$

The above frame is used to introduce a $\overset{˚}{g}$-orthonormal frame $\left\{{\overset{˚}{e}}_{a}\right\}$ with associated cobasis $\left\{{\overset{˚}{\omega }}^{b}\right\}$ so that $\langle {\overset{˚}{\omega }}^{b},{\overset{˚}{e}}_{a}\rangle =\delta_{a}{}^{b}$. This is done by setting

$${\overset{˚}{e}}_{0}\equiv \partial_{\tau },\;{\overset{˚}{e}}_{i}\equiv \frac{1}{\tau }{\overset{˚}{c}}_{i}$$

and

$${\overset{˚}{\omega }}^{0}\equiv \text{d}\tau ,\;{\overset{˚}{\omega }}^{i}=\tau {\overset{˚}{\alpha }}^{i},$$

so that

$$\overset{˚}{g}=\eta_{ab}{\overset{˚}{\omega }}^{a}⊗{\overset{˚}{\omega }}^{b}.$$

#### (ii) The connection coefficients

The connection coefficients ${\overset{˚}{\gamma }}_{i}{}^{k}{}_{j}$ of the Levi–Civita connection $\overset{˚}{D}$ with respect to the frame $\left\{{\overset{˚}{c}}_{i}\right\}$ are defined through the relations

$${\overset{˚}{D}}_{i}{\overset{˚}{c}}_{j}={\overset{˚}{\gamma }}_{i}{}^{k}{}_{j}{\overset{˚}{c}}_{k},\;\gamma_{i}{}^{k}{}_{j}\equiv \langle {\overset{˚}{\alpha }}^{k},{\overset{˚}{D}}_{i}{\overset{˚}{c}}_{j}\rangle .$$

Similarly, for the connection coefficients ${\overset{˚}{\Gamma }}_{i}{}^{k}{}_{j}$ of the Levi–Civita connection $\overset{˚}{\nabla }$ with respect to the frame $\left\{{\overset{˚}{e}}_{a}\right\}$ one has that

$${\overset{˚}{\nabla }}_{a}{\overset{˚}{e}}_{b}={\overset{˚}{\Gamma }}_{a}{}^{c}{}_{b}{\overset{˚}{e}}_{c},\;{\overset{˚}{\Gamma }}_{a}{}^{c}{}_{b}\equiv \langle {\overset{˚}{\omega }}^{c},{\overset{˚}{\nabla }}_{a}{\overset{˚}{e}}_{b}\rangle .$$

Using these relations, it follows that the only non-vanishing connection coefficients are

4.5 $${\overset{˚}{\Gamma }}_{i}{}^{k}{}_{j}=\frac{1}{\tau }{\overset{˚}{\gamma }}_{i}{}^{k}{}_{j},\;{\overset{˚}{\Gamma }}_{i}{}^{j}{}_{0}={\overset{˚}{\chi }}_{i}{}^{j},\;{\overset{˚}{\Gamma }}_{i}{}^{0}{}_{j}=\frac{1}{\tau }\delta_{ij}\;\mathrm{and}\;{\overset{˚}{\Gamma }}_{0}{}^{j}{}_{i}=-\frac{1}{\tau }\delta_{i}{}^{j},$$

where ${\overset{˚}{\chi }}_{i}{}^{j}$ denote the components of the *Weingarten tensor*.

Thus, all the connection coefficients are smooth over $[\tau_{⋆},\infty )\times S$.

#### (iii) Conformal fields

The components of the conformal fields appearing in the extended conformal Einstein field equations are obtained by solving the conformal Einstein constraints discussed in §2c.

This is done by means of an adapted frame with $e_{0}=\partial_{\tau }$ and by making the identification Ω↦Θ in equations (2.8*a*–j). The analysis of these equations gives

4.6*a* $${\overset{˚}{D}}_{i}\Omega =0,\;\overset{˚}{\Sigma }\equiv n(\Theta )=\tau \;\mathrm{and}\;\overset{˚}{s}=1$$

and

4.6*b* $${\overset{˚}{L}}_{i}=0,\;{\overset{˚}{L}}_{ij}=0,\;\overset{˚}{d}{}^{*}{}_{ij}=0\;\mathrm{and}\;\overset{˚}{d}{}_{ij}=0.$$

Thus, all the fields are regular up to the conformal boundary and the metric (4.4) is conformally flat.

### (e) Evolution equations

In this section, we discuss the evolution system associated with the extended conformal Einstein equations (2.5) written in terms of a conformal Gaussian system. In addition, we also discuss the subsidiary evolution system satisfied by the zero-quantities associated to the field equations, (2.4a–d), and the supplementary zero-quantities (2.6*a*–c).

#### (i) The conformal Gaussian gauge

To obtain suitable evolution equations for the conformal fields a *conformal Gaussian gauge* is used. More precisely, it is assumed that a region U⊂M is covered by a congruence of non-intersecting conformal geodesics. Then, by choosing

$$\Theta_{⋆}=\frac{1}{2},\;{\dot{\Theta }}_{⋆}=0\;\mathrm{and}\;{\ddot{\Theta }}_{⋆}=-\frac{1}{2},$$

for $\tau =\tau_{⋆}$, $\tau_{⋆}\in (0,1)$, proposition 2.2 gives the conformal factor

4.7 $$\Theta (\tau )=\frac{1}{2}(1-{\left(\tau -\tau_{⋆}\right)}^{2})$$

along the curves of the congruence. The choice of initial data for the conformal factor is associated to a congruence that leaves orthogonally a fiduciary initial hypersurface $S_{⋆}$ with $\tau =\tau_{⋆}$. Since the conformal factor Θ given by equation (4.7) does not depend on the initial data for the evolution equations it can be regarded as valid not only for the background solution but also for its perturbations. The choice ${\dot{\Theta }}_{⋆}=0$ has the consequence that the Weyl connection is just the conformal Levi–Civita connection on the initial hypersurface $S_{⋆}$.

Along the congruence of conformal geodesics one considers a g-orthogonal frame $\left\{e_{0}\right\}$ which is Weyl-propagated and such that $\tau =e_{0}$. The Weyl connection ${\hat{\nabla }}_{a}$ associated to the congruence then satisfies

$${\hat{\nabla }}_{\tau }e_{a}=0\;\mathrm{and}\;\hat{L}(\tau ,\cdot )=0.$$

By choosing the parameter, τ of the conformal geodesics as time coordinate one gets the additional gauge condition

$$e_{0}=\partial_{\tau }\;\mathrm{and}\;e_{0}{}^{\mu }=\delta_{0}{}^{\mu }.$$

On $S_{⋆}$ we choose some local coordinates $\underline{x}=(x^{\alpha })$. These coordinates can be extended off the initial hypersurface so that the coordinates $\left(\tau ,\underline{x}\right)$ thus obtained are *conformal Gaussian coordinates*.

#### (ii) Structural properties of the evolution and subsidiary equations

In the conformal Gaussian gauge, the various fields associated with the extended vacuum conformal Einstein field equations satisfy the evolution equations

4.8*a* $$\begin{array}{rl} & \partial_{\tau }e_{b}{}^{\nu }=-\hat{\Gamma }{}_{b}{}^{c}{}_{0}e_{c}{}^{\nu }, \end{array}$$

4.8*b* $$\begin{array}{rl} & \partial_{\tau }\hat{L}{}_{db}=\hat{\Gamma }{}_{0}{}^{c}{}_{d}\hat{L}{}_{cb}+\hat{\Gamma }{}_{0}{}^{c}{}_{b}\hat{L}{}_{dc}+d_{a}\hat{d}{}^{a}{}_{b0d}, \end{array}$$

4.8*c* $$\begin{array}{rl} & \partial_{\tau }f_{i}=-f_{j}\hat{\Gamma }{}_{i}{}^{j}{}_{0}+\hat{L}{}_{i0}, \end{array}$$

4.8*d* $$\begin{array}{rl} & \partial_{\tau }(\hat{\Gamma }{}_{b}{}^{c}{}_{d})=-\hat{\Gamma }{}_{f}{}^{c}{}_{d}\hat{\Gamma }{}_{b}{}^{f}{}_{0}-\Xi \hat{d}{}^{c}{}_{d0b}-2{\delta_{d}}^{c}{\hat{L}}_{b0}-2{\delta_{0}}^{c}{\hat{L}}_{bd}+2g_{d0}g^{ce}{\hat{L}}_{be}, \end{array}$$

4.8*e* $$\begin{array}{rl} & \partial_{\tau }d_{bd}+\epsilon^{ef}{}_{(d}D_{f}d^{*}{}_{b)e}=2a_{f}\epsilon_{(d}^{ef}d^{*}{}_{b)e}-\chi d_{bd}+2\chi^{f}{}_{(b}d_{d)f} \end{array}$$

4.8*f* $$\begin{array}{rl} \;\mathrm{and}\; & \partial_{\tau }d^{*}{}_{bd}-\epsilon^{e}{}_{f(d}D^{f}d_{b)e}=2a^{f}{\epsilon_{f(d}}^{e}d_{b)e}-\chi d^{*}{}_{bd}+2\chi_{(b}^{f}d^{*}{}_{d)f}. \end{array}$$

Letting e, Γ, $\hat{L}$ and ϕ denote, respectively, the independent components of the coefficients of the frame, the connection coefficients, the Schouten tensor of the Weyl connection and the rescaled Weyl tensor and setting, for convenience, u≡(υ,ϕ) with υ≡(e,Γ,L) and $\phi \equiv (d,d^{*})$ one has the following:

**Lemma 4.1.**

*The extended conformal Einstein field equations (2.5) expressed in terms of a conformal Gaussian gauge imply that the evolution equations (4.8a)–(4.8f) can be written as a symmetric hyperbolic system for the components* (υ,ϕ) *of the form*

4.9*a* $$\partial \upsilon =K\upsilon +Q(\Gamma )\upsilon +L(\bar{x})\phi$$

*and*

4.9*b* $$(I+A^{0}(e))\partial_{\tau }\phi +A^{\alpha }(e)\partial_{\alpha }\phi =B(\Gamma )\phi ,$$

*where* I *is the unit matrix*, K *is a constant matrix*, Q(Γ) *is a smooth matrix-valued function*, $L(\bar{x})$ *is a smooth matrix-valued function of the coordinates*, $A^{\mu }(e)$ *are Hermitian matrices depending smoothly on the frame coefficients and* B(Γ) *is a smooth matrix-valued function of the connection coefficients*.

Regarding the subsidiary evolution system, it follows from the system

4.10*a* $$\begin{array}{rl} {\hat{\nabla }}_{0}\hat{\Sigma }{}_{b}{}^{d}{}_{c} & \;=-\frac{1}{3}\hat{\Gamma }{}_{c}{}^{e}{}_{0}\hat{\Sigma }{}_{e}{}^{d}{}_{b}-\frac{1}{3}\hat{\Gamma }{}_{c}{}^{e}{}_{0}\hat{\Sigma }{}_{e}{}^{d}{}_{b}-\hat{\Xi }{}^{d}{}_{0bc},\\ {\hat{\nabla }}_{0}\hat{\Xi }{}^{d}{}_{ebc} & \;=\hat{\Gamma }{}_{b}{}^{f}{}_{0}\hat{\Xi }{}^{d}{}_{ecf}+\hat{\Gamma }{}_{c}{}^{f}{}_{0}\hat{\Xi }{}^{d}{}_{efb}\\ & \;\;-\hat{\Sigma }{}_{b}{}^{f}{}_{c}\hat{R}{}^{d}{}_{e0f}-\frac{1}{2}\Theta \epsilon {}^{f}{}_{0bc}\epsilon {}_{e}{}^{dgh}\Lambda {}_{fgh} \end{array}$$

4.10*c* $$\begin{array}{rl} & \;\;+\epsilon {}^{f}{}_{0bc}\delta^{g}d{}^{*d}{}_{efg}+3S{}_{e0}{}^{dg}\hat{\Delta }{}_{cbg},\\ {\hat{\nabla }}_{0}{\hat{\Delta }}_{bcd} & \;=\hat{\Gamma }{}_{b}{}^{e}{}_{0}\hat{\Delta }{}_{ced}+\hat{\Gamma }{}_{c}{}^{e}{}_{0}\hat{\Delta }{}_{ebd}\\ & \;\;-\hat{\Xi }{}^{e}{}_{0bc}\hat{L}{}_{ed}+\delta_{b}d_{e}d{}^{e}{}_{dc0}+\delta_{c}d_{e}d{}^{e}{}_{d0b} \end{array}$$

4.10*e* $$\begin{array}{rl} & \;\;+\Theta \gamma {}_{be}d{}^{e}{}_{dc0}+\Theta \gamma {}_{ce}d{}^{e}{}_{d0b}-\frac{1}{2}\epsilon {}_{0bc}{}^{f}\epsilon {}_{d}{}^{egh}\Lambda {}_{fgh}\beta_{e},\\ \hat{\nabla }{}_{0}\hat{\Omega }{}_{bc} & \;=\hat{\Xi }{}^{e}{}_{[b}{}^{af}d{}_{c]efa}-\hat{\Xi }{}^{e}{}_{f}{}^{af}d{}_{eabc}\\ & \;\;+\frac{1}{2}\hat{\Sigma }{}_{a}{}^{e}{}_{f}\nabla_{e}d{}^{fa}{}_{bc}+\varsigma {}^{fa}d{}_{fabc}-\chi \Omega {}_{bc}, \end{array}$$

4.10*g* $$\begin{array}{rl} {\hat{\nabla }}_{0}\delta_{i} & \;=\gamma_{i0}-\hat{\Gamma }{}_{i}{}^{e}{}_{0}\delta_{e}; \end{array}$$

4.10*h* $$\begin{array}{rl} {\hat{\nabla }}_{0}\gamma_{ic} & \;=-\gamma_{jc}\hat{\Gamma }{}_{i}{}^{j}{}_{0}-\beta_{0}\gamma_{ic}-\beta_{c}\gamma_{i0}+\eta_{0c}(\beta^{e}\gamma_{ie}-2\lambda \Theta^{-2}\delta_{i}) \end{array}$$

4.10*i* $$\begin{array}{rl} {\hat{\nabla }}_{0}\varsigma_{jk} & \;=\hat{\Gamma }{}_{j}{}^{e}{}_{0}\varsigma {}_{ke}+\hat{\Gamma }{}_{k}{}^{e}{}_{0}\varsigma {}_{ej}+\frac{1}{2}{\hat{\Delta }}_{jk0}+\frac{1}{2}\hat{\Xi }{}^{e}{}_{0jk}f_{e}+\frac{1}{2}\hat{\Sigma }{}_{j}{}^{e}{}_{k}\hat{\Gamma }{}_{e}{}^{f}{}_{0}f_{f}, \end{array}$$

that the zero-quantities ${\hat{\Sigma }}_{a}{}^{c}{}_{b}$, ${\hat{\Xi }}^{a}{}_{bcd}$, ${\hat{\Delta }}_{abc}$, ${\hat{\Lambda }}_{abc}$, $\delta_{ab}$, $\gamma_{ab}$ and $\varsigma_{ab}$ satisfy, if the conformal evolution equations (4.8*a*–*e*) hold, a symmetric hyperbolic system which is homogeneous in the zero-quantities. More precisely, upon defining $\hat{X}\equiv (\hat{\Sigma }{}_{a}{}^{c}{}_{b},\hat{\Xi }{}^{c}{}_{dab},\hat{\Delta }{}_{abc},\hat{\Lambda }{}_{abc},\delta_{a},\gamma {}_{ab},\varsigma {}_{ab})$, these equations can be recasted as a symmetric hyperbolic system of the form

4.11 $$\partial_{\tau }\hat{\text{X}}=\text{H}(\hat{\text{X}}),$$

where H(0)=0. The particular situation in which all the zero-quantities vanish identically gives rise to the subsidiary evolution system.

Since the spacetime $\left(\tilde{M},\overset{˚}{\tilde{g}}\right)$ has compact spatial sections there is no need to care about boundaries and the associated difficulties of constraint violations advecting from them, governed through the subsidiary system—see [22,23].

### (f) The perturbative argument

In the following, we look for solutions to the system (4.9*a*,b) of the form

$$\hat{\text{u}}=\overset{˚}{\text{u}}+\overset{˘}{\text{u}}$$

where $\overset{˚}{u}$ is the solution to the conformal evolution equations (4.8*a*–f) implied by a background solution, while $\overset{˘}{u}$ denotes a small perturbation. Accordingly, one can set

4.12*a* $$\hat{\upsilon }=\overset{˚}{\upsilon }+\overset{˘}{\upsilon },\;\hat{\phi }=\overset{˘}{\phi },$$

and

4.12*b* $$\hat{e}=\overset{˚}{e}+\overset{˘}{e},\;\hat{\Gamma }=\overset{˚}{\Gamma }+\overset{ˇ}{\Gamma }.$$

Now, on the initial surface $S_{⋆}$, described by the condition $\tau =\tau_{⋆}$, one has that ${\overset{˚}{u}}_{⋆}=({\overset{˚}{\upsilon }}_{⋆},{\overset{˚}{\phi }}_{⋆})=({\overset{˚}{\upsilon }}_{⋆},0)$ being the exact de Sitter-like solution. As the conformal factor Θ and the covector d are universal, it follows that

$$\partial_{\tau }\overset{˚}{\upsilon }=\text{K}\overset{˚}{\upsilon }+\text{Q}(\overset{˚}{\upsilon },\overset{˚}{\upsilon }).$$

Substituting (4.12*a*,b) into equations (4.9*a*,b) and upon defining the following matrices:

$${\bar{\text{A}}}^{0}(\tau ,\underline{x},\overset{˘}{\text{u}})\equiv \left(\begin{array}{cc} \text{I} & 0\\ 0 & \text{I}+{\text{A}}^{0}(\overset{˚}{e}+\overset{˘}{e}) \end{array}\right)\;\mathrm{and}\;{\bar{\text{A}}}^{\alpha }(\tau ,\underline{x},\overset{˘}{\text{u}})\equiv \left(\begin{array}{cc} 0 & 0\\ 0 & {\text{A}}^{\alpha }(\overset{˚}{e}+\overset{˘}{e}) \end{array}\right)$$

and

$$\bar{\text{B}}(\tau ,\underline{x},\overset{˘}{\text{u}})\equiv \overset{˘}{\text{u}}\bar{\text{Q}}\overset{˘}{\text{u}}+\bar{\text{L}}(\bar{x})\overset{˘}{\text{u}}+\bar{\text{K}}\overset{˘}{\text{u}},$$

where

$$\begin{array}{rl} & \overset{˘}{\text{u}}\bar{\text{Q}}\overset{˘}{\text{u}}\equiv \left(\begin{array}{cc} \overset{˘}{\upsilon }\text{Q}\overset{˘}{\upsilon } & 0\\ 0 & \text{B}(\overset{˘}{\Gamma })\overset{˘}{\phi } \end{array}\right),\;\bar{\text{L}}(\bar{x})\overset{˘}{\text{u}}\equiv \left(\begin{array}{cc} \overset{˚}{\upsilon }\text{Q}\overset{˘}{\upsilon }+\text{Q}(\overset{˘}{\Gamma })\overset{˚}{\upsilon } & \text{L}(\bar{x})\overset{˘}{\phi }\\ 0 & 0 \end{array}\right),\\ \;\mathrm{and}\; & \bar{\text{K}}\overset{˘}{\text{u}}\equiv \left(\begin{array}{cc} \text{K}\overset{˘}{\upsilon } & 0\\ 0 & \text{B}(\overset{˚}{\Gamma })\overset{˘}{\phi } \end{array}\right), \end{array}$$

it is possible to write the evolution equations for $\overset{˘}{u}=(\overset{˘}{\upsilon },\overset{˘}{\phi })$ as a quasi-linear symmetric hyperbolic system

4.13 $${\bar{\text{A}}}^{0}(\tau ,\underline{x},\overset{˘}{\text{u}})\partial_{\tau }\overset{˘}{\text{u}}+{\bar{\text{A}}}^{\alpha }(\tau ,\underline{x},\overset{˘}{\text{u}})\partial_{\alpha }\overset{˘}{\text{u}}=\bar{\text{B}}(\tau ,\underline{x},\overset{˘}{\text{u}}).$$

Existence and stability results for the solution to the initial value problem for the system (5.15) with data $\overset{˘}{u}(0,\underline{x})\equiv {\overset{˘}{u}}_{⋆}(\underline{x})$ follow from known results for symmetric hyperbolic systems over $R^{n}$. More precisely, it is observed that:

(a) The matrices ${\bar{A}}^{\mu }(\tau ,\underline{x},{\overset{˘}{u}}_{⋆})$ are positive definite and depend linearly on the solution $\overset{˘}{u}$ with coefficients which are constant.
(b) The dependence of $\bar{B}$ on $\overset{˘}{u}$ is at most quadratic: there are linear and quadratic terms for the connection coefficients; linear terms for the components of the Schouten tensor. The explicit dependence on $\left(\tau ,\underline{x}\right)$ comes from the conformal factor and the covector $d_{a}$—this dependence is smooth.
(c) The connection coefficients and the components of the Schouten tensor of the background solution are smooth functions ($C^{\infty }$) of $\left(\tau ,\underline{x}\right)$.
(d) The dependence of the frame coefficients of the background solution is smooth ($C^{\infty }$) on τ for $\tau \in [\tau_{⋆},\frac{3}{2}]$ with $\tau_{⋆}\neq 0$.

It follows from the above observations that the conditions of theorem (3.4) are satisfied, thus ensuring existence, uniqueness and Cauchy stability for the solution $\overset{˘}{u}$.

The existence and Cauchy stability of the solution to the initial value problem for the original conformal evolution problem

$${\text{A}}^{0}(\tau ,\underline{x},\hat{\text{u}})\partial_{\tau }\hat{\text{u}}+{\text{A}}^{\alpha }(\tau ,\underline{x},\hat{\text{u}})\partial_{\alpha }\hat{\text{u}}=\text{B}(\tau ,\underline{x},\hat{\text{u}})$$

and

$$\hat{\text{u}}{|}_{⋆}={\overset{˚}{\text{u}}}_{⋆}+{\overset{˘}{\text{u}}}_{⋆}\in H^{m}(S_{⋆},R^{N})\;\mathrm{for}\;m\geq 4$$

follows from the fact that $\hat{u}$ satisfies the same properties as $\overset{˘}{u}$ and then it exists in the same solution manifold and with the same regularity properties, existence and uniqueness.

### (g) A solution to the Einstein field equations

In this section, we discuss the connection between the solution to the conformal evolution systems and the actual solution to the Einstein field equations.

From the discussion in §4e((ii)), it follows that the independent components of the zero-quantities $\hat{X}\equiv (\hat{\Sigma }{}_{a}{}^{c}{}_{b},\hat{\Xi }{}^{c}{}_{dab},\hat{\Delta }{}_{abc},\hat{\Lambda }{}_{abc},\delta_{a},\gamma {}_{ab},\varsigma {}_{ab})$ satisfy the symmetric hyperbolic system (4.11). Then, a solution to the initial value problem

$$\partial_{\tau }\hat{\text{X}}=\text{H}(\hat{\text{X}})$$

and

$${\hat{\text{X}}}_{⋆}=0$$

is given by $\hat{X}=0$. Moreover, from theorem 3.4 it follows that this is the unique solution. Thus, the zero-quantities must vanish on $[\tau_{⋆},1)\times S_{⋆}$. This result is summarized by the following

**Proposition 4.2 (Propagation of the constraints).**

*Let* ${\hat{u}}_{⋆}={\overset{˚}{u}}_{⋆}+{\overset{˘}{u}}_{⋆}$ *denote initial data for the conformal evolution equations on a 3-manifold* $S_{⋆}$ *such that*

$$\hat{\Sigma }{}_{a}{}^{c}{}_{b}{|}_{S_{⋆}}=0,\;\hat{\Xi }{}^{c}{}_{dab}{|}_{S_{⋆}}=0,\;\hat{\Delta }{}_{abc}{|}_{S_{⋆}}=0\;\mathrm{and}\;{\hat{\Lambda }}_{abc}{|}_{S_{⋆}}=0,$$

*and*

$$\delta {}_{a}{|}_{S_{⋆}}=0,\;\gamma {}_{ab}{|}_{S_{⋆}}=0\;\mathrm{and}\;\varsigma {}_{ab}{|}_{S_{⋆}}=0,$$

*then the solution* $\overset{˘}{u}$ *to the conformal evolution equations implies a* $C^{m-2}$ *solution* $\hat{u}=\overset{˚}{u}+\overset{˘}{u}$ *to the extended conformal field equations on* $[\tau_{⋆},1)\times S_{⋆}$.

Now, given the propagation of the constraints, proposition 4.2, and proposition 2.1 it follows that the metric $g=\Theta^{2}\tilde{g}$ obtained from the solution to the conformal evolution equations implies a solution to the vacuum Einstein field equations with λ=3.

The main result of this discussion is contained in the following theorem

**Theorem 4.3.**

*Let* ${\hat{u}}_{⋆}={\overset{˚}{u}}_{⋆}+{\overset{˘}{u}}_{⋆}$ *denote smooth initial data for the conformal evolution equations satisfying the conformal constraint equations on a hypersurface* $S_{⋆}$. *Then, there exists* ε>0 *such that if*

$$||{\overset{˘}{\text{u}}}_{⋆}|{|}_{S_{⋆},m}<\varepsilon ,\;m\geq 4$$

*then there exists a unique* $C^{m-2}$ *solution* $\tilde{g}$ *to the vacuum Einstein field equation with positive Cosmological constant over* $[{\tilde{\tau }}_{⋆},1)\times S_{⋆}$ *for* $\tau_{⋆}>0$ *whose restriction to* $S_{⋆}$ *implies the initial data* ${\hat{u}}_{⋆}$. *Moreover, the solution* $\hat{u}$ *remains suitably close to the background solution* $\overset{˚}{u}$.

## 5. Schwarzschild–de Sitter spacetimes

In this section, the behaviour of the conformal geodesics in the Cosmological region of the sub-extremal Schwarzschild–de Sitter spacetime is discussed. The aim of this analysis is to adapt the technique described in the de Sitter-like setting and valid, in general, for asymptotically simple spacetimes to the black hole case as presented in [16].

### (a) Basic properties

The *Schwarzschild–de Sitter spacetime* $\left(\tilde{M},\overset{˚}{\tilde{g}}\right)$ is a spherically symmetric solution to the vacuum Einstein field equations with positive Cosmological constant (2.1) with $\tilde{M}=R\times R^{+}\times S^{2}$ and line element given in *standard coordinates* (t,r,θ,φ) by

5.1 $$\overset{˚}{\tilde{g}}=-\left(1-\frac{2m}{r}-\frac{\lambda }{3}r^{2}\right)\text{d}t⊗\text{d}t+{\left(1-\frac{2m}{r}-\frac{\lambda }{3}r^{2}\right)}^{-1}\text{d}r⊗\text{d}r+r^{2}\sigma ,$$

where

$$\sigma \equiv \text{d}\theta ⊗\text{d}\theta +\sin^{2}\theta \text{d}\varphi ⊗\text{d}\varphi ,$$

denotes the standard metric on $S^{2}$. The coordinates (t,r,θ,φ) take the range

t∈(−∞,∞),r∈(0,∞),θ∈(0,π)andφ∈[0,2π).

This line element can be rescaled so that

5.2 $$\overset{˚}{\tilde{g}}=-D(r)\;\text{d}t⊗\text{d}t+\frac{1}{D(r)}\text{d}r⊗\text{d}r+r^{2}\sigma ,$$

where

$$D(r)\equiv 1-\frac{M}{r}-r^{2}\;\mathrm{and}\;M\equiv 2m\sqrt{\frac{\lambda }{3}}.$$

In our conventions, M, r and λ are dimensionless quantities.

### (b) Horizons and global structure

The location of the horizons of the Schwarzschild–de Sitter spacetime follows from the analysis of the zeros of the function D(r) in the line element (5.2).

Since λ>0, the function D(r) can be factorized as

$$D(r)=-\frac{1}{r}(r-r_{b})(r-r_{c})(r-r_{-}),$$

where $r_{b}$ and $r_{c}$ are, in general, distinct positive roots of D(r) and $r_{-}$ is a negative root. Moreover, one has that

$$0<r_{b}<r_{c},\;r_{c}+r_{b}+r_{-}=0.$$

The root $r_{b}$ corresponds to a black hole type of horizon and $r_{c}$ to a Cosmological de Sitter-like type of horizon. Using Cardano’s formula for cubic equations, we have

5.3*a* $$\begin{array}{rl} & r_{-}=-\frac{2}{\sqrt{3}}\cos\left(\frac{\phi }{3}\right), \end{array}$$

5.3*b* $$\begin{array}{rl} & r_{b}=\frac{1}{\sqrt{3}}\left(\cos\left(\frac{\phi }{3}\right)-\sqrt{3}\sin\left(\frac{\phi }{3}\right)\right) \end{array}$$

5.3*c* $$\begin{array}{rl} \;\mathrm{and}\; & r_{c}=\frac{1}{\sqrt{3}}\left(\cos\left(\frac{\phi }{3}\right)+\sqrt{3}\sin\left(\frac{\phi }{3}\right)\right). \end{array}$$

where the parameter ϕ is defined through the relation

5.4 $$M=\frac{2\cos\phi }{3\sqrt{3}},\;\phi \in \left(0,\frac{\pi }{2}\right).$$

**Figure 2.**
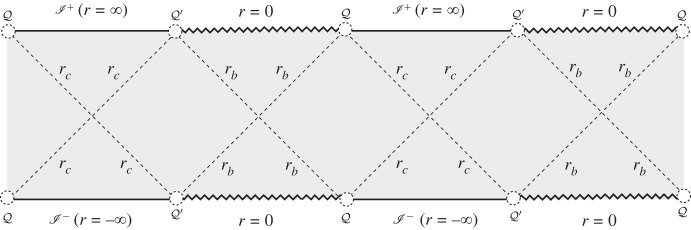
Penrose–Carter diagram of the sub-extremal Schwarzschild–de Sitter spacetime. The serrated line denotes the location of the singularity; the continuous black line denotes the conformal boundary; the dashed line shows the location of the black hole and Cosmological horizons denoted by $H_{b}$ and $H_{c}$ respectively. As described in the main text, these horizons are located at $r=r_{b}$ and $r=r_{c}$. The excluded points Q and $Q^{\prime }$ where the singularity seems to meet the conformal boundary correspond to asymptotic regions of the spacetime that does not belong to the singularity nor the conformal boundary.

The sub-extremal case is characterized by $0<M<2/3\sqrt{3}$ and ϕ∈(0,π/2), describing a black hole in a Cosmological setting. The Penrose–Carter diagram of the sub-extremal Schwarzschild–de Sitter is well known—see figure 2.

### (c) Construction of a conformal Gaussian gauge in the Cosmological region

This study begins with the qualitative analysis of the behaviour of the conformal geodesics of the Schwarzschild–de Sitter spacetime prescribed in terms of data on hypersurfaces of constant r in the Cosmological region.

#### (i) Basic set-up

It is assumed that

$$r_{c}<r<\infty$$

corresponding to the Cosmological region of the Schwarzschild–de Sitter spacetime. Given a fixed $r=r_{⋆}$, $S_{⋆}$ denotes the space-like hypersurface of constant $r=r_{⋆}$ in this region. Points on $S_{⋆}$ are described in terms of the coordinates (t,θ,φ).

In order to fix the congruence of conformal geodesics, the value of the conformal factor $\Theta_{⋆}$ over $S_{⋆}$ is chosen so that

$$\Theta_{⋆}=1\;\mathrm{and}\;{\dot{\Theta }}_{⋆}=0.$$

The second condition implies that the resulting conformal factor will have a time reflection symmetry with respect to $S_{⋆}$. Then it is required that

$${\tilde{x}}_{⋆}^{\prime }⊥S_{⋆}\;\mathrm{and}\;{\tilde{\beta }}_{⋆}=\Theta_{⋆}^{-1}\;d\Theta_{⋆}.$$

The latter, in turn, implies that

5.5 $$t=t_{⋆}\;t{}^{\prime }{}_{⋆}=\frac{1}{\sqrt{D_{⋆}}},\;r{}^{\prime }{}_{⋆}=0,\;{\tilde{\beta }}_{t⋆}=0\;\mathrm{and}\;{\tilde{\beta }}_{r⋆}=0.$$

These conditions give rise to a congruence of conformal geodesics which acts trivially in the angular directions. Accordingly, the analysis of these curves is effectively given by the metric

5.6 $$\tilde{\ell }=-D(r)\;\text{d}t⊗\text{d}t+\frac{1}{D(r)}\text{d}r⊗\text{d}r.$$

Finally, in order to exclude the asymptotic points Q and $Q^{\prime }$, it is defined

$$R_{∙}=\{p\in S_{⋆}\;|\;t(p)\in (-t_{∙},t_{∙})\},$$

where the constant $t_{∙}$ is assumed large enough so that $D^{+}(R_{∙})\cap I^{+}\neq \emptyset$.

#### (ii) Analysis of the behaviour of the conformal geodesics

The congruence of conformal geodesics prescribed by the initial data (5.5) is such that $\beta^{2}=0$, so that after reparametrization reduces to a congruence of metric geodesics. Thus, the geodesic equations imply that

5.7 $$r^{\prime }=\sqrt{\gamma^{2}-D(r)}\;\mathrm{and}\;D(r)t^{\prime }{}^{2}-\frac{1}{D(r)}r^{\prime 2}=1,$$

where γ is a constant. Evaluating at $S_{⋆}$ one readily finds that

$$t_{⋆}^{\prime }=\frac{|\gamma |}{|D_{⋆}|},$$

with $D_{⋆}<0$. Moreover, since the units normal to $S_{⋆}$ and ${\tilde{x}}_{⋆}^{\prime }$ are parallel to each other then γ=0.

In order to study the behaviour of these curves and obtain simpler expressions, it is set λ=3 and $\tau_{⋆}=0$. It follows then from proposition (2.2) that the conformal factor is

5.8 $$\Theta (\tau )=1-\frac{1}{4}\tau^{2}.$$

Now, since the relation between the physical proper time $\tilde{\tau }$ and the unphysical proper time τ is obtained from equation (2.13) so that

5.9 $$\tilde{\tau }=2\;\mathrm{arctanh}\left(\frac{\tau }{2}\right)\;\mathrm{and}\;\tau =2\tanh\left(\frac{\tilde{\tau }}{2}\right),$$

then

$$\tau \to \pm 2,\;\mathrm{as}\;\tilde{\tau }\to \pm \infty$$

and since this congruence of conformal geodesics is reparametrized as metric geodesics, it will reach the conformal boundary orthogonally [14]. Now, since the dependence of the physical proper time $\tilde{\tau }$ on r is given by

$$\tilde{\tau }=\int_{r_{⋆}}^{r}\sqrt{\frac{\bar{r}}{\left(\bar{r}-r_{b})(\bar{r}-r_{c})(\bar{r}-r_{-}\right)}}\;d\bar{r}$$

which can be written in terms of elliptic functions (e.g. [24]), it follows from the general theory of elliptic functions that $\tilde{\tau }(r,r_{⋆})$ is an analytic function of its arguments. Moreover, one has that

$$\tilde{\tau }\to \infty \;\text{as}\;r\to \infty .$$

Accordingly, the curves escape to infinity in an infinite amount of physical proper time. Using the reparametrization formulae (5.9) the latter corresponds to a finite amount of unphysical proper time.

#### (iii) Analysis of the behaviour of the conformal deviation equation

In [18] (see also [19]), it has been shown that for congruences of conformal geodesics in spherically symmetric spacetimes the behaviour of the deviation vector of the congruence can be understood by considering the evolution of a scalar $\tilde{\omega }$ satisfying the equation

5.10 $$\text{⧸}D_{{\tilde{x}}^{\prime }}\text{⧸}D_{{\tilde{x}}^{\prime }}\tilde{\omega }=\left(\beta^{2}+\frac{1}{2}R[\tilde{\ell }]\right)\tilde{\omega }+\text{⧸}D_{\tilde{z}}\beta ,$$

where ⧸D denotes the Levi–Civita covariant derivative of $\tilde{\ell }$ and $R[\tilde{\ell }]$ denotes the Ricci scalar of $\tilde{\ell }$. If $\tilde{\omega }$ does not vanish, then the congruence is non-intersecting.

Since in the present case one has β=0 and $R[\tilde{\ell }]=-\partial_{r}^{2}D(r)$, it follows that the evolution equation (5.10) takes the form

$$\frac{d^{2}\tilde{\omega }}{d{\tilde{\tau }}^{2}}=\left(1+\frac{M}{r^{3}}\right)\tilde{\omega },\;r\equiv r(\tilde{\tau },r_{⋆}).$$

Since this setting $r\geq r_{⋆}>r_{c}$ and $\omega \equiv \Theta \tilde{\omega }$, it follows that

$$\frac{d^{2}\bar{\omega }}{d{\tilde{\tau }}^{2}}=\bar{\omega },\;\bar{\omega }(0,\rho_{⋆})=\frac{r_{⋆}}{\rho_{⋆}}\;\mathrm{and}\;{\bar{\omega }}^{\prime }(0,\rho_{⋆})=0.$$

By solving this last differential equation and reverting to ω, one has that

$$\omega \geq \frac{r_{⋆}}{\rho_{⋆}}\left(1+\frac{\tau^{2}}{4}\right)>0,$$

which is non-vanishing in the limit τ→±2. Thus, we have the following Proposition

**Proposition 5.1.**

*The congruence of conformal geodesics given by the initial conditions (5.5) leaving the initial hypersurface* $S_{⋆}$ *reach the conformal boundary* $I^{+}$ *without developing caustics*.

**Figure 3.**
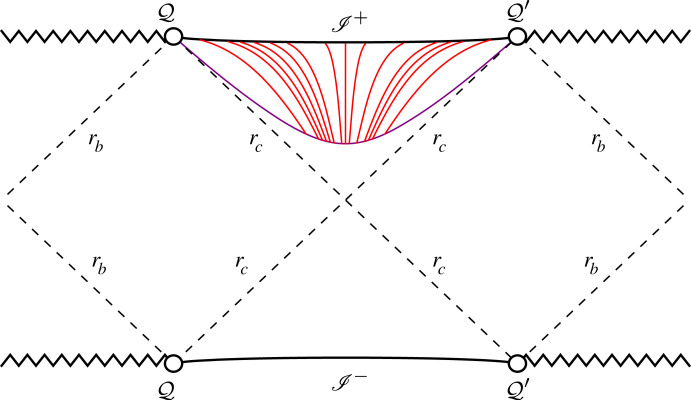
The conformal geodesics are plotted on the Penrose–Carter diagram of the Cosmological region of the sub-extremal Schwarzschild–de Sitter spacetime. The purple line represents the initial hypersurface $S_{⋆}$ corresponding to $r=r_{⋆}$. The red lines represent conformal geodesics with constant time leaving this initial hypersurface.

This behaviour of the conformal geodesics is shown in figure 3.

#### (iv) Conformal Gaussian coordinates in the sub-extremal Schwarzschild–de Sitter spacetime

The congruence of conformal geodesics defined by the initial conditions (5.5) is used to construct a *conformal Gaussian coordinate system* in a domain in the chronological future of $R_{∙}$ containing a portion of the conformal boundary $I^{+}$. This analysis is carried out by considering the coordinate z≡1/r in terms of which the line element (5.2) takes the form

$$\overset{˚}{\tilde{g}}=\frac{1}{z^{2}}\left(-F(z)\;\text{d}t⊗\text{d}t+\frac{1}{F(z)}\text{d}z⊗\text{d}z+\sigma \right),$$

where

$$F(z)\equiv z^{2}D\left(\frac{1}{z}\right).$$

The above expression suggest defining an *unphysical metric* $\bar{g}$ via

$$\bar{g}=\Xi^{2}\overset{˚}{\tilde{g}},\;\Xi \equiv z.$$

More precisely, one has

5.11 $$\bar{g}=-F(z)\text{d}t⊗\text{d}t+\frac{1}{F(z)}\text{d}z⊗\text{d}z+\sigma .$$

Now, let ${\tilde{SdS}}_{I}$ denote the Cosmological region of the Schwarzschild–de Sitter spacetime—that is

$${\tilde{SdS}}_{I}=\{p\in \tilde{M}\;|\;r(p)>r_{c}\}.$$

Moreover, denote by $SdS_{I}$ the conformal representation of ${\tilde{SdS}}_{I}$ defined by the conformal factor Θ defined by the non-singular congruence of conformal geodesics. Let z≡1/r, for $z<z_{c}$ one has that in terms of these coordinates

5.12 $$SdS_{I}=\{p\in R\times R\times S^{2}\;|\;0\leq z(p)\leq z_{⋆}\},$$

where $z_{⋆}\equiv 1/r_{⋆}$ with $r_{⋆}>r_{c}$.

The conformal geodesics defined by the initial conditions (5.5) define a map ψ which is analytic in the parameters $\left(\tau ,t_{⋆}\right)$. This map is invertible since the Jacobian of the transformation is non-zero for the given value of the parameters. The inverse map

$$\psi^{-1}:[0,z_{⋆}]\times [-t_{∙},t_{∙}]\to [0,2]\times [-t_{∙},t_{∙}],\;(t,z)\mapsto (\tau (t,z),t_{⋆}(t,z))$$

gives the transformation from the *standard Schwarzschild coordinates* (t,z,θ,φ) into the *conformal Gaussian coordinates* $\left(\tau ,t_{⋆},\theta ,\varphi \right)$. This result is summarized by the following

**Proposition 5.2.**

*The congruence of conformal geodesics on* $SdS_{I}$ *defined by the initial conditions on* $S_{⋆}$ *given by (5.5) induce a conformal Gaussian coordinate system over* $D^{+}(R_{∙})$ *which is related to the standard coordinates* (t,r) *via a map which is analytic*.

### (d) The background spacetime as a solution to the conformal Einstein field equations

The Schwarzschild–de Sitter spacetime in the region

$$M_{∙}\equiv [0,2]\times [-t_{∙},t_{∙}]$$

is cast as a solution to the extended conformal Einstein field equations by means of a Weyl propagated frame.

#### (i) The frame

Since the congruence of conformal geodesics implied by the initial data (5.5) satisfies $\tilde{\beta }=0$, the Weyl propagation equation (2.10) reduces to the usual parallel propagation equation. Given the spherical symmetry of the Schwarzschild–de Sitter spacetime, the discussion of a frame adapted to the symmetry of the spacetime can be carried out by considering the two-dimensional Lorentzian metric (5.6). The *time leg* of the frame is set as $e_{0}=\dot{x}$ so that

$$e_{0}=\Theta^{-1}{\tilde{x}}^{\prime },$$

where ${\tilde{x}}^{\prime }={\tilde{t}}^{\prime }\partial_{t}+{\tilde{r}}^{\prime }\partial_{r}$. Now, upon defining

$$\omega \equiv \epsilon_{\ell }({\tilde{x}}^{\prime },\cdot )$$

with $\langle \omega ,{\tilde{x}}^{\prime }\rangle =0$, one has that the *radial leg* of the frame is given by

$$e_{1}=\Theta \omega^{♯}.$$

The Weyl propagated frame $\left\{e_{a}\right\}$ is completed by choosing *two arbitrary orthonormal vectors* ${\tilde{e}}_{2⋆}$ and ${\tilde{e}}_{3⋆}$ spanning the tangent space of $S^{2}$ and defining the vectors $\left\{e_{2},e_{3}\right\}$ on $M_{∙}$ by constantly extending the value of the associated coefficients along the conformal geodesics. This analysis leads to the following result

**Proposition 5.3.**

*Let* ${\tilde{x}}^{\prime }$ *denote the vector tangent to the conformal geodesics defined by the initial data (5.5) and let* $\left\{e_{2⋆},e_{3⋆}\right\}$ *be an arbitrary orthonormal pair of vectors spanning the tangent bundle of* $S^{2}$. *Then the frame* $\left\{e_{0},e_{1},e_{2},e_{3}\right\}$ *obtained by the procedure described in the previous paragraph is a* g-*orthonormal Weyl propagated frame. The frame depends analytically on the unphysical proper time* τ and the initial position $t_{⋆}$ *of the curve*.

#### (ii) The Weyl connection

The connection coefficients associated to a conformal Gaussian gauge are made up of two pieces: the 1-form defining the Weyl connection and the Levi–Civita connection of the metric $\bar{g}$.

The congruence of conformal geodesics discussed in §5c arises from initial data chosen so that the curves with tangent given by ${\tilde{x}}^{\prime }$ satisfy the standard (affine) geodesic equation. Consequently, the (spatial) 1-form $\tilde{\beta }$ vanishes. Now, since ${\tilde{x}}^{\prime }=r^{\prime }\partial_{r}$, by observing equation (5.7) for $r^{\prime }$ with γ=0 and by introducing z=1/r, it follows that

$$\beta \approx -\frac{1}{z}\text{d}z\;\text{for}\;z\approx 0.$$

Then, from the conformal transformation rule

$$\bar{\beta }=\beta +\Xi^{-1}\text{d}\Xi$$

and by recalling that Ξ=z, it follows that $\bar{\beta }$ vanishes at $I^{+}$. However, $\bar{\beta }\neq 0$ away from the conformal boundary.

#### (iii) The connection coefficients

Since the coordinates and connection coefficients associated with the physical connection $\tilde{\nabla }$ are not well adapted to a discussion near the conformal boundary we resort to the unphysical Levi–Civita connection $\bar{\nabla }$ to compute $\hat{\nabla }$.

The connection coefficients ${\hat{\Gamma }}_{a}{}^{b}{}_{c}$ are defined through the relation

$${\hat{\nabla }}_{a}e_{c}={\hat{\Gamma }}_{a}{}^{b}{}_{c}e_{b}.$$

The only non-vanishing Christoffel symbols ${\bar{\Gamma }}_{\mu }{}^{\nu }{}_{\lambda }$ are given by

$$\begin{array}{rl} & {\bar{\Gamma }}_{t}{}^{t}{}_{z}=-{\bar{\Gamma }}_{z}{}^{z}{}_{z}=\frac{z((3/2)Mz-1)}{1+z^{2}(Mz-1)},\\ & {\bar{\Gamma }}_{t}{}^{z}{}_{t}=z\left(\frac{3}{2}Mz-1\right)(1+z^{2}(Mz-1))\\ \;\mathrm{and}\; & {\bar{\Gamma }}_{\varphi }{}^{\theta }{}_{\varphi }=-\cos\theta \sin\theta \;\mathrm{and}\;{\bar{\Gamma }}_{\theta }{}^{\varphi }{}_{\varphi }=\cot\theta . \end{array}$$

These coefficients are analytic at z=0. Since a contraction with the coefficients of the frame does not change this, it follows that the Weyl connection coefficients ${\hat{\Gamma }}_{a}{}^{b}{}_{c}$ are smooth functions of the coordinates used in the conformal Gaussian gauge on the future of the fiduciary initial hypersurface $S_{⋆}$ up to and beyond the conformal boundary.

#### (iv) The rescaled Weyl tensor

Given a time-like vector, the components of the rescaled Weyl tensor $d_{abcd}$ can be encoded in the electric and magnetic parts relative to the given vector. For the vector ${\bar{e}}_{0}$ these are given by

$$d_{ac}=d_{abcd}{\bar{e}}_{0}{}^{b}{\bar{e}}_{0}{}^{d}\;\mathrm{and}\;d^{*}{}_{ac}=d{}^{*}{}_{abcd}{\bar{e}}_{0}{}^{b}{\bar{e}}_{0}{}^{d},$$

where $d{}^{*}{}_{abcd}$ denotes the Hodge dual of $d_{abcd}$. A computation using the package xAct for Mathematica [25] readily gives that the only non-zero components of the electric part are given by

$$\begin{array}{rl} & d_{tt}=-M(z^{2}(1-Mz)-1),\\ & d_{\theta \theta }=-\frac{M}{2}\\ \;\mathrm{and}\; & d_{\varphi \varphi }=-\frac{M}{2}\sin^{2}\theta , \end{array}$$

while the magnetic part vanishes identically. These expressions are regular at z=0—by disregarding the coordinate singularity due to the use of spherical coordinates. The smoothness of the components of the Weyl tensor is retained when contracted with the coefficients of the frame $\left\{e_{a}\right\}$.

#### (v) The Schouten tensor

A similar computer algebra calculation shows that the non-zero components of the Schouten tensor of the metric $\bar{g}$ are given by

$$\begin{array}{rl} & {\bar{L}}_{tt}=\frac{1}{2}(2Mz-1)(1+z^{2}(Mz-1)),\\ & {\bar{L}}_{zz}=-\frac{1}{2}\frac{\left(2Mz-1\right)}{1+z^{2}(Mz-1)},\\ & {\bar{L}}_{\theta \theta }=-\frac{1}{2}(Mz-1)\\ \;\mathrm{and}\; & {\bar{L}}_{\varphi \varphi }=-\frac{1}{2}\sin^{2}\theta (Mz-1). \end{array}$$

The above expressions are analytic on $M_{∙}$—in particular at z=0 and by disregarding the coordinate singularity on the angular components. To obtain the components of the Schouten tensor associated with the Weyl connection $\hat{\nabla }$ we make use of the transformation rule

$${\bar{L}}_{ab}-{\hat{L}}_{ab}={\bar{\nabla }}_{a}{\bar{\beta }}_{b}-\frac{1}{2}S_{ab}{}^{cd}{\bar{\beta }}_{c}{\bar{\beta }}_{d}.$$

The smoothness of ${\bar{\beta }}_{a}$ has already been established in §5d((ii)). Thus, the components of ${\hat{L}}_{ab}$ with respect to the Weyl propagated frame $\left\{e_{a}\right\}$ are regular on $M_{∙}$.

#### (vi) Construction of a background solution with compact spatial sections

From the previous discussion, it follows that the sub-extremal Schwarzschild–de Sitter spacetime expressed in terms of a conformal Gaussian gauge system gives rise to a solution to the extended conformal Einstein field equations on the region $M_{∙}\subset D^{+}(R_{∙})$. Since $R_{∙}\subset S_{⋆}$ has the topology of $I\times S^{2}$ where I⊂R is an open interval, the spacetime arising from $R_{∙}$ will have spatial sections with the same topology. As part of the perturbative argument is based on the general theory of symmetric hyperbolic systems as given in theorem 3.4 it is convenient to consider solutions with compact spatial sections.

**Figure 4.**
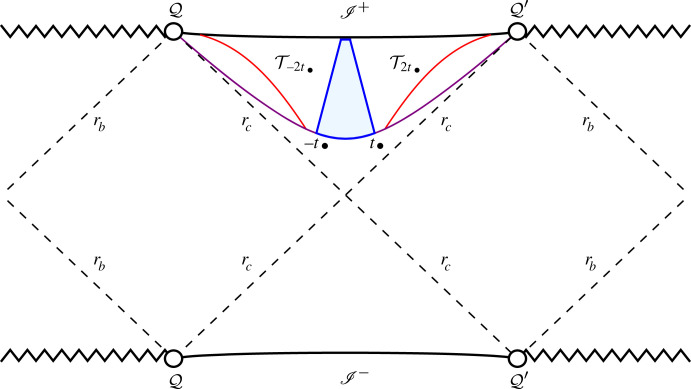
The red curves identify the time-like hypersurfaces $T_{-2t_{∙}}$ and $T_{2t_{∙}}$. The resulting spacetime manifold ${\bar{M}}_{∙}$ has compact spatial sections, ${\bar{S}}_{z}$, with the topology of $S^{1}\times S^{2}$.

This construction is based on the observation that the Killing vector $\xi =\partial_{t}$ in the Cosmological region of the spacetime is space-like. Thus, given a fixed $z_{\circ }<z_{c}$, the hypersurface $S_{z_{\circ }}$ defined by the condition $z=z_{\circ }$ has a translational invariance. Now, one identifies the time-like hypersurfaces $T_{-2t_{∙}}$ and $T_{2t_{∙}}$ generated, respectively, by the future-directed geodesics emanating from $S_{⋆}$ at the points with $t=-2t_{∙}$ and $t=2t_{∙}$ to obtain a smooth spacetime manifold ${\bar{M}}_{∙}$ with compact spatial sections—see figure 4. The metric $\bar{g}$ on $SdS_{I}$ induces a metric on ${\bar{M}}_{∙}$ which, on an abuse of notation, is denoted again by $\bar{g}$. Since the initial conditions defining the congruence of conformal geodesics of §5c have translational invariance, the resulting curves also have this property. Accordingly, the congruence of conformal geodesics on $SdS_{I}$ induces a non-intersecting congruence of conformal geodesics on ${\bar{M}}_{∙}$. Thus, the solution to the extended conformal Einstein field equations in a conformal Gaussian gauge implies a similar solution over the manifold ${\bar{M}}_{∙}$ denoted by $\overset{˚}{u}$. The initial data induced by $\overset{˚}{u}$ on ${\bar{S}}_{⋆}$ will be denoted by ${\overset{˚}{u}}_{⋆}$.

### (e) Structural properties of the evolution and subsidiary equations

The conformal Gaussian gauge system leads to a *hyperbolic reduction* of the extended conformal Einstein field equation (2.5). The particular form of the resulting evolution equations is not required in this analysis, only general structural properties.

The extended conformal Einstein field equations (2.5) expressed in terms of a conformal Gaussian gauge imply evolution equations in the form of a symmetric hyperbolic system for the components υ≡(e,Γ,L) and $\phi \equiv (d,d^{*})$ as in lemma 4.1. Now, since the evolution equations hold, the independent components of the zero-quantities

$${\hat{\Sigma }}_{a}{}^{b}{}_{c},\;{\hat{\Xi }}^{c}{}_{dab},\;{\hat{\Delta }}_{abc},\;\Lambda_{abc},\;\delta_{a},\;\gamma_{ab}\;\mathrm{and}\;\varsigma_{ab},$$

not determined by either the evolution equations or the gauge conditions satisfy a symmetric hyperbolic system which is homogeneous in the zero-quantities. As a result, if the zero-quantities vanish on a fiduciary space-like hypersurface ${\bar{S}}_{⋆}$, then they also vanish on the domain of dependence [3].

### (f) The perturbative argument

Let $\hat{u}\equiv (\hat{v},\hat{\phi })$ and $\overset{˚}{u}$ denotes the *background solution* being a solution to the evolution equations arising from the initial data ${\overset{˚}{u}}_{⋆}$ prescribed on ${\bar{S}}_{⋆}$. Solutions to the evolution equations which can be regarded as a perturbation of the background solution are constructed by introducing a perturbative argument

$$\hat{\text{u}}=\overset{˚}{\text{u}}+\overset{˘}{\text{u}}$$

with $\overset{˘}{u}$ being a small perturbation. This means, in particular, that one can write

5.13 $$\hat{e}=\overset{˚}{e}+\overset{˘}{e},\;\hat{\Gamma }=\overset{˚}{\Gamma }+\overset{˘}{\Gamma }\;\mathrm{and}\;\hat{\phi }=\overset{˚}{\phi }+\overset{˘}{\phi }.$$

The components of $\overset{˘}{e}$, $\overset{˘}{\Gamma }$ and $\overset{˘}{\phi }$ are our unknowns. Making use of the decomposition (5.13) and exploiting that $\overset{˚}{u}$ is a solution to the conformal evolution equations one obtains the equations

5.14*a* $$\partial_{\tau }\overset{˘}{\upsilon }=\text{K}\overset{˘}{\upsilon }+\text{Q}(\overset{˚}{\Gamma }+\overset{˘}{\Gamma })\overset{˘}{\upsilon }+\text{Q}(\overset{˘}{\Gamma })\overset{˚}{\upsilon }+\text{L}(\bar{x})\overset{˘}{\phi }+\text{L}(\bar{x})\overset{˚}{\phi },$$

and

5.14*b* $$(\text{I}+{\text{A}}^{0}(\overset{˚}{e}+\overset{˘}{e}))\partial_{\tau }\overset{˘}{\phi }+{\text{A}}^{\alpha }(\overset{˚}{e}+\overset{˘}{e})\partial_{\alpha }\overset{˘}{\phi }=\text{B}(\overset{˚}{\Gamma }+\overset{˘}{\Gamma })\overset{˘}{\phi }+\text{B}(\overset{˚}{\Gamma }+\overset{˘}{\Gamma })\overset{˚}{\phi }.$$

Now, it is convenient to define

$${\bar{\text{A}}}^{0}(\tau ,\underline{x},\overset{˘}{\text{u}})\equiv \left(\begin{array}{cc} \text{I} & 0\\ 0 & \text{I}+{\text{A}}^{0}(\overset{˚}{e}+\overset{˘}{e}) \end{array}\right)\;\mathrm{and}\;{\bar{\text{A}}}^{\alpha }(\tau ,\underline{x},\overset{˘}{\text{u}})\equiv \left(\begin{array}{cc} 0 & 0\\ 0 & {\text{A}}^{\alpha }(\overset{˚}{e}+\overset{˘}{e}) \end{array}\right),$$

and

$$\bar{\text{B}}(\tau ,\underline{x},\overset{˘}{\text{u}})\equiv \overset{˘}{\text{u}}\bar{\text{Q}}\overset{˘}{\text{u}}+\bar{\text{L}}(\bar{x})\overset{˘}{\text{u}}+\bar{\text{K}}\overset{˘}{\text{u}},$$

where

$$\begin{array}{rl} & \overset{˘}{\text{u}}\bar{\text{Q}}\overset{˘}{\text{u}}\equiv \left(\begin{array}{cc} \overset{˘}{\upsilon }\text{Q}\overset{˘}{\upsilon } & 0\\ 0 & \text{B}(\overset{˘}{\Gamma })\overset{˘}{\phi }+\text{B}(\overset{˘}{\Gamma })\overset{˚}{\phi } \end{array}\right),\;\bar{\text{L}}(\bar{x})\overset{˘}{\text{u}}\equiv \left(\begin{array}{cc} \overset{˚}{\upsilon }\text{Q}\overset{˘}{\upsilon }+\text{Q}(\overset{˘}{\Gamma })\overset{˚}{\upsilon } & \text{L}(\bar{x})\overset{˘}{\phi }+\text{L}(\bar{x})\overset{˚}{\phi }\\ 0 & 0 \end{array}\right)\\ \;\mathrm{and}\; & \bar{\text{K}}\overset{˘}{\text{u}}\equiv \left(\begin{array}{cc} \text{K}\overset{˘}{\upsilon } & 0\\ 0 & \text{B}(\overset{˚}{\Gamma })\overset{˘}{\phi }+\text{B}(\overset{˚}{\Gamma })\overset{˚}{\phi }, \end{array}\right) \end{array}$$

denote, respectively, expressions which are quadratic, linear and constant terms in the unknowns.

In terms of the above expressions, it is possible to rewrite the system (5.14*a*,b) in the more concise form

5.15 $${\bar{\text{A}}}^{0}(\tau ,\underline{x},\overset{˘}{\text{u}})\partial_{\tau }\overset{˘}{\text{u}}+{\bar{\text{A}}}^{\alpha }(\tau ,\underline{x},\overset{˘}{\text{u}})\partial_{\alpha }\overset{˘}{\text{u}}=\bar{\text{B}}(\tau ,\underline{x},\overset{˘}{\text{u}}).$$

This is a quasi-linear symmetric hyperbolic system for which theorem 3.4 can be applied to obtain an existence and stability result for a Cauchy problem with initial data ${\overset{˘}{u}}_{⋆}$.

### (g) A solution to the Einstein field equations

The evolution equations (5.14*a*,b) imply the same subsidiary system as for de Sitter-like spacetimes. Thus, the *propagation of the constraints* follows from the same argument—see proposition 3.1 §4g. Given the propagation of the constraints and proposition 2.1, one has the metric $g=\Theta^{2}\tilde{g}$ obtained from the solution to the conformal evolution equations implies a solution to the vacuum Einstein field equations with positive Cosmological constant on $\tilde{M}\equiv D^{+}(R_{∙})$.

The main result of this discussion is contained in the following theorem:

**Theorem 5.4.**

*Let* ${\hat{u}}_{⋆}={\overset{˚}{u}}_{⋆}+{\overset{˘}{u}}_{⋆}$ *denote smooth initial data for the conformal evolution equations satisfying the conformal constraint equations on a hypersurface* ${\bar{S}}_{⋆}$. *There exists* ε>0 *such that if*

$$||{\overset{˘}{\text{u}}}_{⋆}|{|}_{{\bar{S}}_{⋆},m}<\varepsilon ,\;m\geq 4$$

*then there exists a unique* $C^{m-2}$ *solution* $\tilde{g}$ *to the vacuum Einstein field equation with positive Cosmological constant over* $[{\tilde{\tau }}_{⋆},\infty )\times {\bar{S}}_{⋆}$ *for* ${\tilde{\tau }}_{⋆}>0$ *whose restriction to* ${\bar{S}}_{⋆}$ *implies the initial data* ${\hat{u}}_{⋆}$. *Moreover, the solution* $\hat{u}$ *remains suitably close to the background solution* $\overset{˚}{u}$.

In particular, the resulting spacetime $\left(\tilde{M},\tilde{g}\right)$ is a nonlinear perturbation of the sub-extremal Schwarzschild–de Sitter spacetime on a portion of the Cosmological region of the background solution which contains a portion of the asymptotic region.

## 6. Conclusion

This review article provides a discussion based on [15,16] describing how the extended conformal Einstein field equations and a gauge adapted to the conformal geodesics can be used to study the evolution of vacuum spacetimes with positive Cosmological constant. In the de Sitter-like case, this analysis identifies a class of spacetimes for which it is possible to prove nonlinear stability and the existence of a regular conformal representation. More precisely, a class of de Sitter-like spacetimes is identified which can be conformally embedded into a portion of a cylinder whose spatial sections have negative scalar curvature. The conformal embedding is realized by means of a conformal factor Θ which depends quadratically on the affine parameter τ of the conformal geodesics and this parameter is also used as a time coordinate for the physical metric. This result led to the idea that this technique could be adapted to black hole type of spacetimes. When adapting the strategy to the Cosmological region of the Schwarzschild–de Sitter spacetime, one encounters several difficulties. Whether in the de Sitter-like case, the explicit form of the unphysical metric $\overset{˚}{g}$ is known. In the Schwarzschild–de Sitter case the explicit form of the unphysical metric g is not known. This means that, in the former case, one can solve the conformal Einstein constraint equations and recast the unphysical spacetime $\left(M,\overset{˚}{g}\right)$ as a solution to the conformal Einstein field equations. Moreover, one can construct a conformal Gaussian gauge system and show how the main and subsidiary evolution systems can be recasted as quasi-linear symmetric hyperbolic systems. In the latter case, since the unphysical metric g is not known, one analyses the behaviour of a congruence of $\tilde{g}$-conformal geodesics to use their properties to construct the conformal Gaussian gauge system. The background solution is obtained by computing the components of the curvature tensors in the conformal Levi–Civita connection and then using the transformation laws to recast them as solutions to the extended conformal Einstein field equations. The main evolution system is of the same form as for the de Sitter-like spacetime with the difference that for the Schwarzschild–de Sitter spacetime whereas the magnetic part of the rescaled Weyl tensor vanishes, the electric part of this tensor is non-vanishing. As a consequence, the main evolution system contains extra terms involving $\overset{˚}{\phi }$. Nonetheless, once recasted as a symmetric hyperbolic system its structural form is the same as for the de Sitter-like spacetime. For what concerns the subsidiary evolution system, it is the same as for the de Sitter-like spacetime. Another difference is that the sub-extremal Schwarzschild–de Sitter spacetime expressed in terms of a conformal Gaussian gauge system gives rise to a solution to the extended conformal Einstein field equations on the region $M_{∙}$. This region corresponds to the future domain of dependence of a portion $R_{∙}$ of the initial hypersurface $S_{⋆}$ with topology $I\times S^{2}$ where I⊂R. Thus, one needs to recast this solution as a solution with compact spatial sections in order to use theorem 3.4 to prove its existence, uniqueness and stability. As a result, one shows that it is possible to construct solutions to the vacuum Einstein field equations in this region containing a portion of the asymptotic region and which are nonlinear perturbations of the exact Schwarzschild–de Sitter spacetime. Crucially, although the spacetimes constructed have an infinite extent to the future, they exclude the *asymptotic points* Q and $Q^{\prime }$. From the analysis of the asymptotic initial value problem in [17], it is known that these points contain singularities of the conformal structure. Thus, they cannot be dealt with by the approach discussed in the article. In order to have a complete statement on the nonlinear stability of the Cosmological region it is necessary to address the asymptotic points. Moreover, since the initial hypersurfaces $S_{⋆}$ considered in the article are space-like and the evolution doesn’t include the Cosmological horizon $r_{c}$. A complete statement should also include the case in which $r=r_{c}$. This suggests reformulating the existence and stability results in [16] in terms of a characteristic initial value problem with data prescribed on Cosmological horizons. Again, to avoid the singularities of the conformal structure, the characteristic data has to be prescribed away from the asymptotic points. Alternatively, one could consider datasets which become exactly Schwarzschild–de Sitter near the asymptotic points. The associated evolution problem by means of a generalization of the methods used in [26] should allow us to reach any suitable hypersurface with constant r.

## Data Availability

This article has no additional data.

## Footnotes

1 The value λ=3 for the Cosmological constant is conventional and set for convenience. This analysis can be carried out for any other positive value of λ.
